# Competition for calnexin binding regulates secretion and turnover of misfolded GPI-anchored proteins

**DOI:** 10.1083/jcb.202108160

**Published:** 2023-09-13

**Authors:** Amber M. Cheatham, Nishi Raj Sharma, Prasanna Satpute-Krishnan

**Affiliations:** 1 Department of Biochemistry and Molecular Biology, Uniformed Services University of the Health Sciences, Bethesda, MD, USA

## Abstract

In mammalian cells, misfolded glycosylphosphatidylinositol (GPI)-anchored proteins (GPI-APs) are cleared out of the ER to the Golgi via a constitutive and a stress-inducible pathway called RESET. From the Golgi, misfolded GPI-APs transiently access the cell surface prior to rapid internalization for lysosomal degradation. What regulates the release of misfolded GPI-APs for RESET during steady-state conditions and how this release is accelerated during ER stress is unknown. Using mutants of prion protein or CD59 as model misfolded GPI-APs, we demonstrate that inducing calnexin degradation or upregulating calnexin-binding glycoprotein expression triggers the release of misfolded GPI-APs for RESET. Conversely, blocking protein synthesis dramatically inhibits the dissociation of misfolded GPI-APs from calnexin and subsequent turnover. We demonstrate an inverse correlation between newly synthesized calnexin substrates and RESET substrates that coimmunoprecipitate with calnexin. These findings implicate competition by newly synthesized substrates for association with calnexin as a key factor in regulating the release of misfolded GPI-APs from calnexin for turnover via the RESET pathway.

## Teaser

Misfolded GPI-APs are released from the ER to the Golgi for rapid degradation in lysosomes by the constitutively active and stress-inducible RESET pathway. Using multiple complementary approaches, the authors demonstrate that competition by nascent glycoproteins for calnexin-binding promotes the release of misfolded GPI-APs for RESET.

## Introduction

Secretory pathway protein homeostasis (proteostasis) is fundamental to cellular and organismal health. Secretory pathway substrates including transmembrane, soluble, and glycosylphosphatidylinositol (GPI) anchored proteins (GPI-APs) are synthesized, folded, and processed in the endoplasmic reticulum (ER) prior to secretion to the cell surface (Kinoshita et al., 2013). The central dilemma in this process is that protein folding is inherently error prone and occasionally generates toxic misfolded proteins (Dobson, 2003; Princiotta et al., 2003). To maintain optimal cellular function, the ER chaperones and enzymes, cumulatively referred to as “ER protein quality control (PQC),” monitor protein folding and dispose misfolded products (Braakman and Hebert, 2013; Ellgaard and Helenius, 2003; McCaffrey and Braakman, 2016; Sun and Brodsky, 2019). By helping incoming proteins fold for eventual secretion or by discarding terminally misfolded proteins, ER PQC maintains the dynamic equilibrium of proteins within the ER.

Major ER clearance pathways, including ER-associated degradation (ERAD), ER autophagy (ER-phagy), and other ER-to-lysosome-associated-degradation (ERLAD) pathways, prevent misfolded secretory or membrane proteins from accessing the cell surface. The ERAD pathways generally involve p97-mediated retrotranslocation of misfolded proteins from the ER to the cytosol for degradation by the ubiquitin-proteasome system (Christianson and Ye, 2014; Ruggiano et al., 2014), while ER-phagy and other ERLAD pathways comprise vesicular transport directly from the ER to lysosomes for degradation, typically through the involvement of lipidated LC3 (De Leonibus et al., 2019; Fregno and Molinari, 2019; Oikonomou and Hendershot, 2020; Wilkinson, 2020). By contrast, mounting evidence suggests that misfolded GPI-APs, including human disease mutants of prion protein (PrP), are not efficiently degraded at the ER by the ERAD or ER-phagy pathways but undergo ER-export to the Golgi prior to lysosomal degradation (Ashok and Hegde, 2009; Fujita et al., 2006; Guo et al., 2020; Lemus et al., 2021; Satpute-Krishnan et al., 2014; Sikorska et al., 2016; Zavodszky and Hegde, 2019). ER-export to the Golgi for downstream lysosomal degradation occurs constitutively (Lemus et al., 2021; Satpute-Krishnan et al., 2014) and during ER stress (Satpute-Krishnan et al., 2014; Zavodszky and Hegde, 2019). Furthermore, in mammalian cells, misfolded GPI-APs gain access to the cell surface during constitutive and ER-stress-induced turnover (Satpute-Krishnan et al., 2014; Zavodszky and Hegde, 2019), which may provide insight into the oft-reported extracellular amyloid aggregates of prion protein detected in the brain tissue of a patient with prion disease (Jeffrey et al., 1994; Scheckel and Aguzzi, 2018). Yet how misfolded substrates are released from the ER is not well understood.

In a previous study, we uncovered a new Tmp21-dependent pathway for the ER-export of misfolded GPI-APs to the Golgi in mammalian cells using PrP* as a model substrate. PrP* is a quantitatively misfolding mutant of prion protein, PrP C179A. Although this ER-clearance pathway is constitutively active, we observed that various ER stressors, including the new expression of PrP* in naïve cells, increase the flux through this pathway (Satpute-Krishnan et al., 2014). Thus, we referred to this ER-to-Golgi clearance pathway as RESET (for Rapid ER Stress-induced Export) to indicate that ER-stress rapidly induces ER-export of misfolded proteins through this pathway (Satpute-Krishnan et al., 2014). Following RESET, misfolded GPI-APs traffic from the Golgi to the cell surface where they are rapidly internalized and directed to lysosomes for destruction. Importantly, this ER-export pathway plays a major role in the steady-state turnover of diverse, unrelated misfolded GPI-APs, including prion disease-associated mutants (Satpute-Krishnan et al., 2014).

In this present study, we investigate what regulates the release of misfolded GPI-APs from the ER for RESET during normal steady-state conditions and how this release is accelerated during acute upregulation of new ER PQC substrates. Our data strongly suggest that calnexin (CNX), an ER-localized chaperone that aids in the folding of newly synthesized N-linked glycoproteins (Hebert et al., 1997; Trombetta and Helenius, 1998; Ware et al., 1995), is a key ER-retention factor for misfolded GPI-APs. Acute degradation of CNX causes a rapid release of misfolded GPI-APs for RESET, while binding of diverse substrates to CNX promotes the release of misfolded GPI-APs from CNX for ER-export during steady-state conditions. Furthermore, we demonstrate a correlation between the load of substrates binding to CNX and the Tmp21-dependent release of misfolded GPI-APs for ER-export. Our results fit with a model that competition for CNX-binding plays a key role in regulating the flux of misfolded GPI-APs moving through the RESET pathway.

## Results

**Figure S1.**
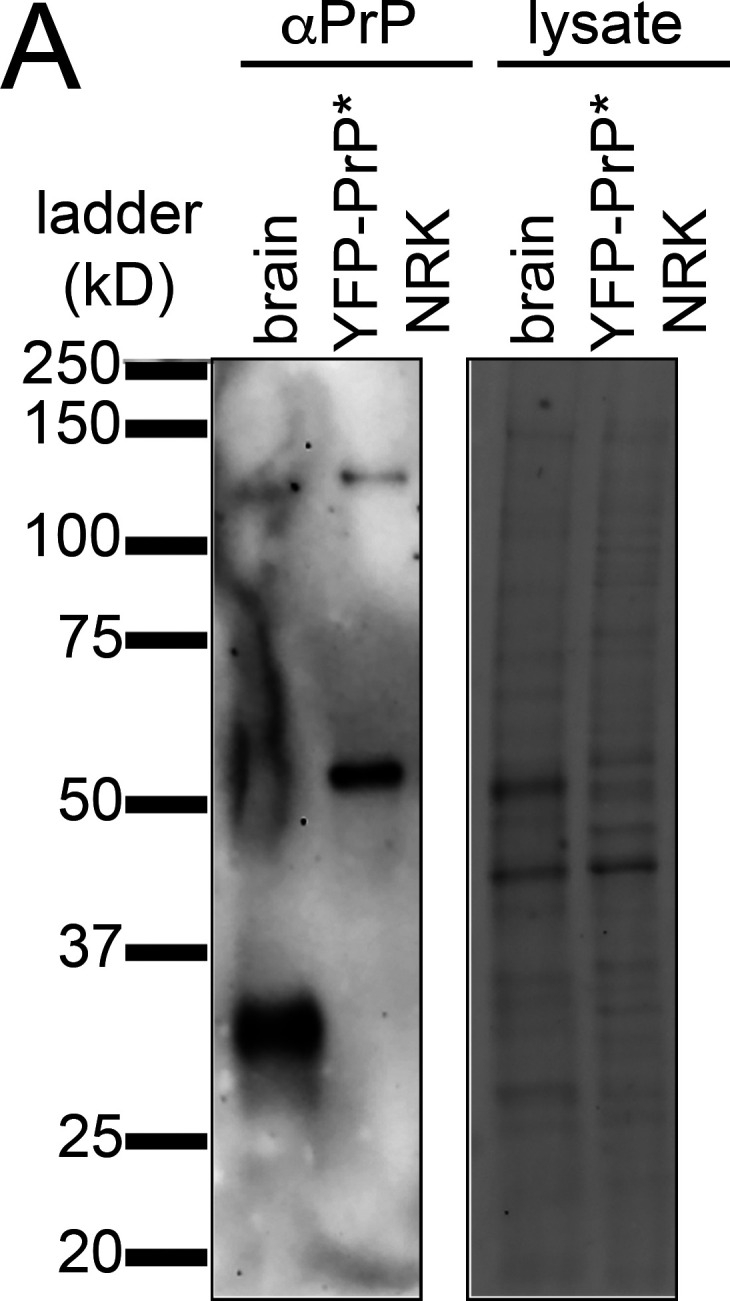
Western blot of endogenous PrP in mouse brain lysate versus stably and exogenously expressed YFP-PrP* in YFP-PrP* NRK cell lysate. (A) Western blot probed with anti-PrP antibody (left panel) and stain-free imaging of total lysate (right panel). Source data are available for this figure: SourceData FS1.

We generated normal rat kidney (NRK) cells that stably express YFP-PrP* (YFP-PrP* NRK cells) and sorted them for low expression levels of YFP-PrP*, below the expression level of endogenous prion protein in mouse brain tissue (Fig. S1 A). N2a cells are long-established as a model system for PrP folding, trafficking, and QC studies (Butler et al., 1988; Krance et al., 2020; Pineau and Sim, 2021; Race et al., 1987; Solassol et al., 2003; Vilette, 2008). However, NRK cells are more adherent to the coverslip and relatively immobile in comparison to N2a cells, facilitating time-lapse imaging over long periods of time as previously demonstrated (Hailey et al., 2010; Satpute-Krishnan et al., 2014). Critically, we established that YFP-PrP* is handled by the RESET pathway in multiple unrelated cell culture model systems including N2a cells (Satpute-Krishnan et al., 2014).

### Blocking global protein translation inhibits the degradation of RESET substrates

**Figure 1.**
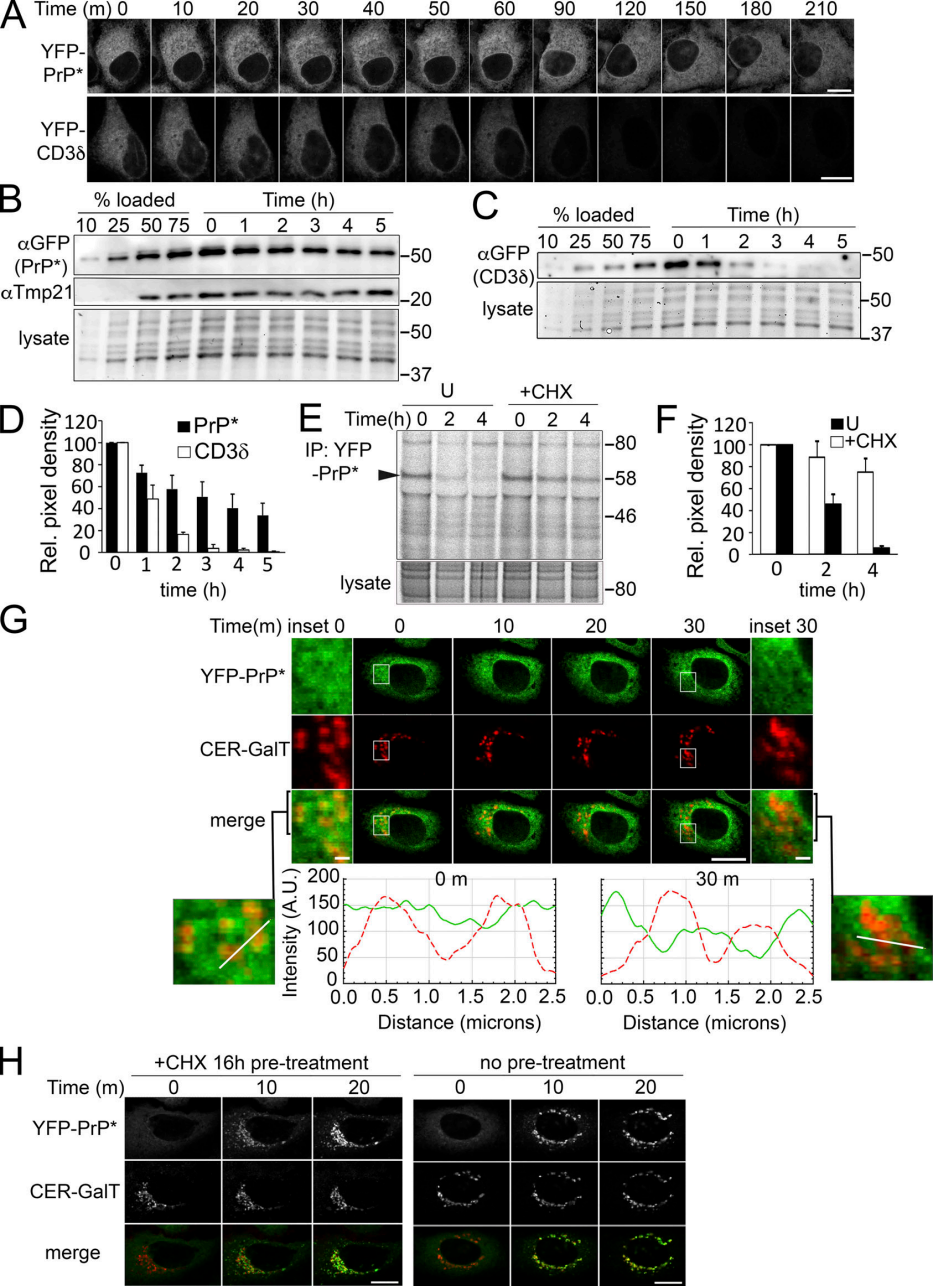
Cycloheximide inhibits steady-state ER-export and degradation of YFP-PrP*. (A) Time-lapse imaging of representative YFP-PrP* NRK or YFP-CD3δ NRK stable transfectants upon treatment with 50 μg/ml cycloheximide. Scale bar, 10 μm. **(B and C)** Representative western blots of cycloheximide-chase assays of (B) YFP-PrP* (probed with anti-GFP antibody) and Tmp21 or (C) YFP-CD3δ (probed with anti-GFP antibody) from stably transfected NRK cells. Cells were collected at the indicated time points after cycloheximide treatment. An equal number of stably transfected cells were seeded in a 12-well dish 24 h prior to cycloheximide treatment. Total input panels labeled “lysates” depict Bio-Rad Stain-free gel imaging system to visually confirm equal loading of total cellular lysates across the time points. Experiments were performed in triplicate. **(D)** Relative pixel densities of YFP-PrP* (black bars) and YFP-CD3δ (white bars) bands of the western blots from the cycloheximide-chase assays, as shown in representative examples in B and C. Western blots of cell lysates were probed with anti-GFP antibody to detect YFP-PrP* and YFP-CD3δ. Quantifications of the YFP-PrP* or YFP-CD3δ bands were performed by creating a rectangular bounding box or region of interest (ROI) that surrounded and enclosed the baseline *t* = 0 band and then dragging the same bounding box from lane to lane to measure the integrated density of the GFP bands from *t* = 0 through *t* = 5 h. Integrated density values were background subtracted and normalized against the loading controls. Loading control values were obtained by measuring the integrated density signal across each lysate lane, instead of individual bands. The normalized density values were measured against the *t* = 0 value to obtain the relative pixel densities, where *t* = 0 is normalized to 1. Bar graph represents the average relative pixel density of three independently performed experiments (*n* = 3, biological replicates). Error bars represent SD. **(E)** Autoradiograph of steady-state chase of YFP-PrP* NRK cells that were left untreated “U” or treated with cycloheximide “+CHX.” Arrowhead is positioned at the molecular weight of the YFP-PrP* band. Anti-GFP immunoprecipitations to capture the YFP-PrP* were performed on cell lysates from YFP-PrP* NRK cells that were radiolabeled 12 h with 35S-labeled cysteine and methionine and chased with non-radioactive medium without “U” (for untreated) or with cycloheximide “+CHX” for 0, 2, or 4 h. **(F)** Quantification of the relative pixel density of the YFP-PrP* bands from triplicate steady-state chases of YFP-PrP* isolated from YFP-PrP* NRK cells that were left untreated “U” (black bars) or treated with cycloheximide “+CHX” (white bars). Quantifications of the radiolabeled YFP-PrP* bands were performed by creating a rectangular bounding box or region of interest (ROI) that surrounded and enclosed the baseline *t* = 0 band, and then dragging the same bounding box to enclose and measure the integrated density of the *t* = 2 and *t* = 4 h bands. Integrated density values were background subtracted, normalized against the intensity across the corresponding lysate lanes. The normalized density values were measured against the *t* = 0 value to obtain the relative pixel densities where *t* = 0 is normalized to 1 for untreated or +cycloheximide “+CHX” samples. Bar graph represents the average relative pixel density of three independently performed experiments (*n* = 3, biological replicates). Error bars represent SD. **(G)** Time-lapse imaging of a representative stably transfected YFP-PrP* and Cerulean (CER)-GalT NRK cell that was treated with CHX. CER-GalT marks the Golgi. Insets are magnified for 0 and 30 m time points. YFP-PrP* and CER-GalT intensity plot profiles of the lines drawn across the 0 and 30 m images are shown as a solid green line (YFP-PrP*) and dashed red line (CER-GalT). Intensity units are arbitrary (A.U.). Scale bar in the main figure is 10 μm, while scale bars in the insets are 1 μm. **(H)** Time-lapse imaging of a representative YFP-PrP* NRK cell that was treated with 1 μM thapsigargin after 16 h of cycloheximide “+CHX 16 h pre-treatment” (left panel) or after no pretreatment (right panel). Source data are available for this figure: SourceData F1.

**Figure S2.**
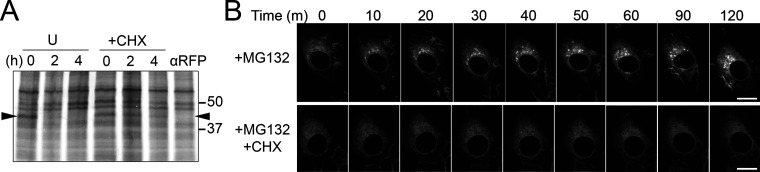
**Degradation of GFP-CD59 C94S via the RESET pathway is inhibited by cycloheximide-treatment. (A)** Autoradiograph of steady-state chase of eGFP-CD59 C94S NRK cells that were left untreated (U) or treated with CHX. Arrowhead is positioned at the molecular weight of eGFP-CD59 C94S. Anti-RFP indicates a negative control where the immunoprecipitation was performed with anti-RFP antibody instead of anti-GFP. **(B)** Time-lapse imaging of representative NRK cells stably expressing GFP-CD59 C94S after treatment with MG132 alone or MG132 + CHX. Scale bar, 10 μm. Source data are available for this figure: SourceData FS2.

We initiated our investigation into the regulation of RESET substrate turnover by comparing the degradation rate of YFP-PrP* with that of the well-characterized ERAD substrate, YFP-CD3δ (Chen et al., 1988). We monitored YFP-PrP* and YFP-CD3δ degradation after cycloheximide-treatment using either time-lapse imaging of YFP-fluorescence (Fig. 1 A) or western blotting for the YFP-tag (Fig. 1, B and C). Cycloheximide-chase assays indicated that YFP-PrP* is a much longer-lived protein quality control substrate with a half-life of ∼3 h as compared with YFP-CD3δ, whose half-life was confirmed to be ∼1 h (Fig. 1 D), as previously reported for CD3δ (Blount et al., 2012; Chen et al., 2014; Schulze et al., 2005). The cycloheximide-chase result for YFP-PrP* did not match the half-life of 1.5 h that we previously determined by radioactive steady-state chase analysis (Satpute-Krishnan et al., 2014). One possible explanation was that cycloheximide treatment itself somehow inhibited protein degradation of RESET substrates. To test this, we performed steady-state chase analysis in untreated or cycloheximide-treated cells. We discovered that adding cycloheximide at the start of the chase did indeed strongly inhibit the degradation of YFP-PrP* (Fig. 1, E and F) and the unrelated RESET substrate, GFP-CD59 C94S (Fig. S2 A). Confocal imaging analysis of the YFP-PrP* in cells that were coexpressing a Golgi marker revealed that within 30 m of cycloheximide treatment, the steady-state Golgi-localized population of YFP-PrP* disappeared, while the ER-localized population of YFP-PrP* remained (Fig. 1 G). This observation indicated that cycloheximide somehow reduced ER-export of YFP-PrP*.

To explain how cycloheximide reduced ER-export of YFP-PrP*, we initially hypothesized that cycloheximide inhibited the replenishment of critical labile factor(s) required for ER-export. Since we previously demonstrated Tmp21 to be an essential RESET factor required for ER-export of YFP-PrP* for RESET (Satpute-Krishnan et al., 2014), we specifically probed the YFP-PrP* blot from the cycloheximide chase assay for Tmp21 (Fig. 1 B). Tmp21 levels remained stable over 5 h of cycloheximide treatment, excluding it as a “labile” ER-export factor. Thus, we took an alternate approach.

We had previously demonstrated that dissociation from ER chaperone CNX and, concomitantly, synchronized release of YFP-PrP* for RESET are induced by thapsigargin (Satpute-Krishnan et al., 2014). Although it is established that thapsigargin is a fast-acting and irreversible SERCA pump inhibitor that allows for the rapid depletion of ER calcium levels (Thastrup et al., 1990) and causes structural alterations in Ca^2+^-binding chaperones CNX (Thammavongsa et al., 2005) and BiP (Preissler et al., 2020), the precise mechanism by which thapsigargin disrupts the ER chaperone(s)–RESET substrate interaction(s) is unknown (discussed below). However, if RESET of YFP-PrP* were dependent on a labile ER-export factor, then extensive treatment times with cycloheximide would allow for the depletion of this labile factor and thereby prevent thapsigargin-induced release of YFP-PrP* for RESET. We found that even after 16 h of cycloheximide pretreatment, thapsigargin still efficiently triggered the release of YPF-PrP* from the ER, demonstrating that the RESET pathway was still intact and discounting the idea of a requisite “labile” factor for the ER-export of RESET substrates (Fig. 1 H).

We devised a new hypothesis to explain how the translation inhibitor cycloheximide dramatically reduced steady-state ER-export and turnover of YFP-PrP*. Considering that cells have a limited chaperone protein folding capacity leading to the continuous release of a subset of the proteome for steady-state turnover (Powers et al., 2009) and our previously published observation that induction of new PrP* increases the flux of PrP* undergoing ER-export (Satpute-Krishnan et al., 2014), we hypothesized that during steady-state conditions, newly synthesized PQC substrates may directly or indirectly compete with misfolded GPI-APs for binding to a limited, steady-state pool of ER-resident chaperones. This competition would ultimately cause a subset of misfolded GPI-APs to be released for the RESET pathway and subsequent lysosomal degradation. Thus, the flux of misfolded GPI-APs through the RESET pathway would correlate with the rate of new protein synthesis.

### The expression of ER PQC substrates induces the release of RESET substrates for ER-export

**Figure 2.**
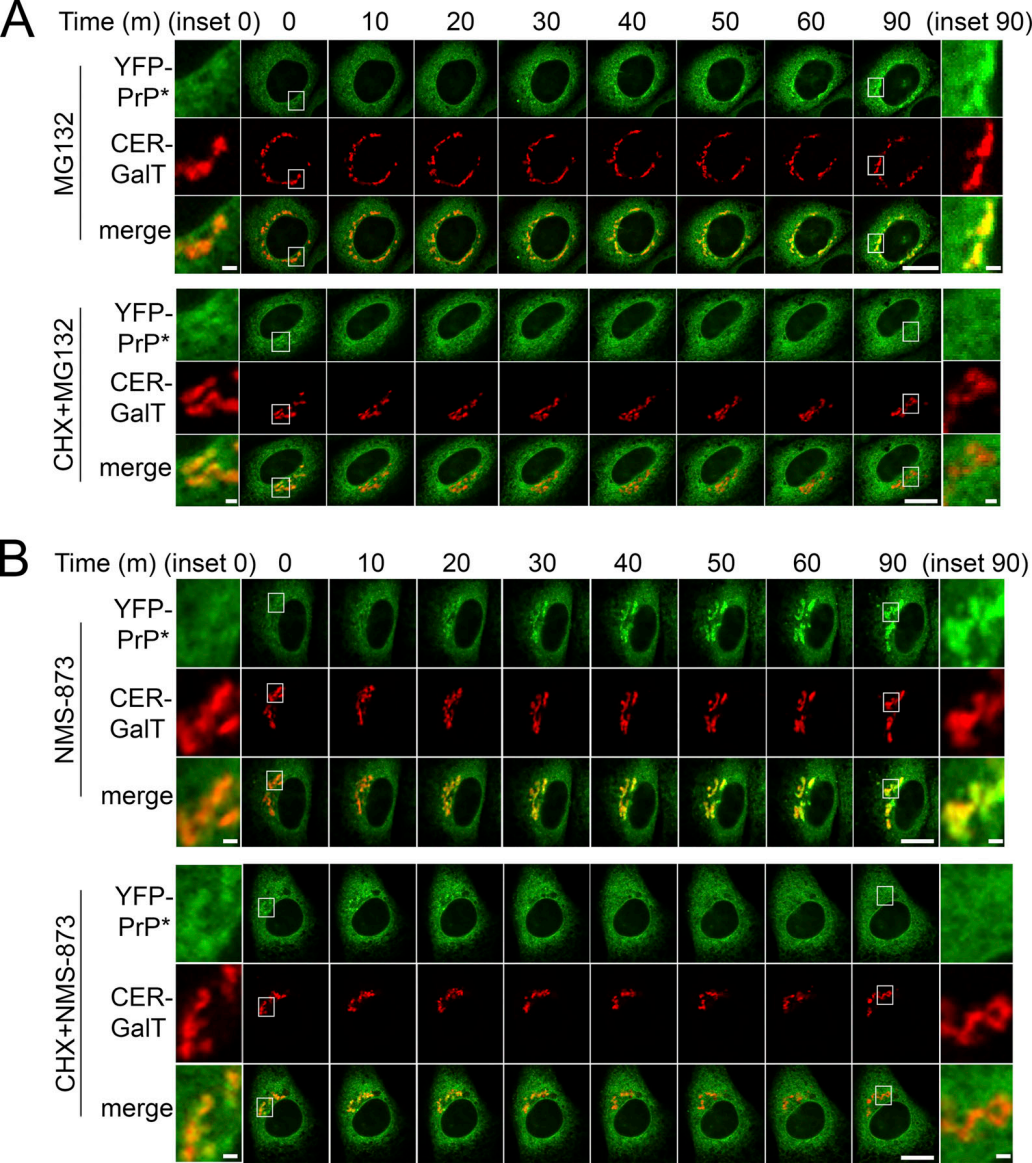
Inhibiting proteasomal degradation increases the flux of YFP-PrP* through the RESET pathway by a mechanism that requires constitutive protein translation. (A and B) Time-lapse imaging of representative YFP-PrP* and Cerulean (CER)-GalT NRK cells after treatment with (A) 10 μM MG132 alone (MG132) or MG132 with 50 μg/ml cycloheximide (CHX+MG132), or (B) 10 μM NMS-873 alone (NMS-873) or NMS-873 with CHX (CHX+NMS-873). CER-GalT is a Golgi marker. Insets are magnified for 0 and 90 min time points. Scale bars in the main figures are 10 μm, while scale bars in the insets are 1 μm.

To test the idea that newly synthesized PQC substrates are able to compete with RESET substrates for ER-resident chaperone binding, we treated YFP-PrP* NRK cells with MG132. MG132 is a proteasome inhibitor that effectively blocks ERAD, allowing misfolded proteins that were destined for degradation to build up in the ER (Feige and Hendershot, 2013; Gelman et al., 2002; Obeng et al., 2006; St-Pierre et al., 2012) and potentially compete for binding to existing ER-chaperones. Indeed, MG132 treatment immediately caused a subtle ER-to-Golgi shift in the localization of a fraction of YFP-PrP*, revealing an increase in the flux of YFP-PrP* moving through the RESET pathway (Fig. 2 A). Cycloheximide, however, blocked the MG132-induced shift in YFP-PrP* localization, which is consistent with the idea that a buildup of newly synthesized PQC substrates promotes RESET of YFP-PrP* (Fig. 2 A). Similarly, MG132 induced RESET of a small fraction of GFP-CD59 C94S, and this effect was completely inhibited in the presence of cycloheximide (Fig. S2 B). Additionally, we treated cells with NMS-873 to block ERAD by inhibiting VCP/p97 (Magnaghi et al., 2013), a AAA ATPase that is integral for retrotranslocation of ERAD substrates (Ye et al., 2001, 2005). RESET of YFP-PrP* was induced by NMS-873-treatment alone, but not when YFP-PrP* NRK cells were treated with both cycloheximide and NMS-873 (Fig. 2 B).

**Figure 3.**
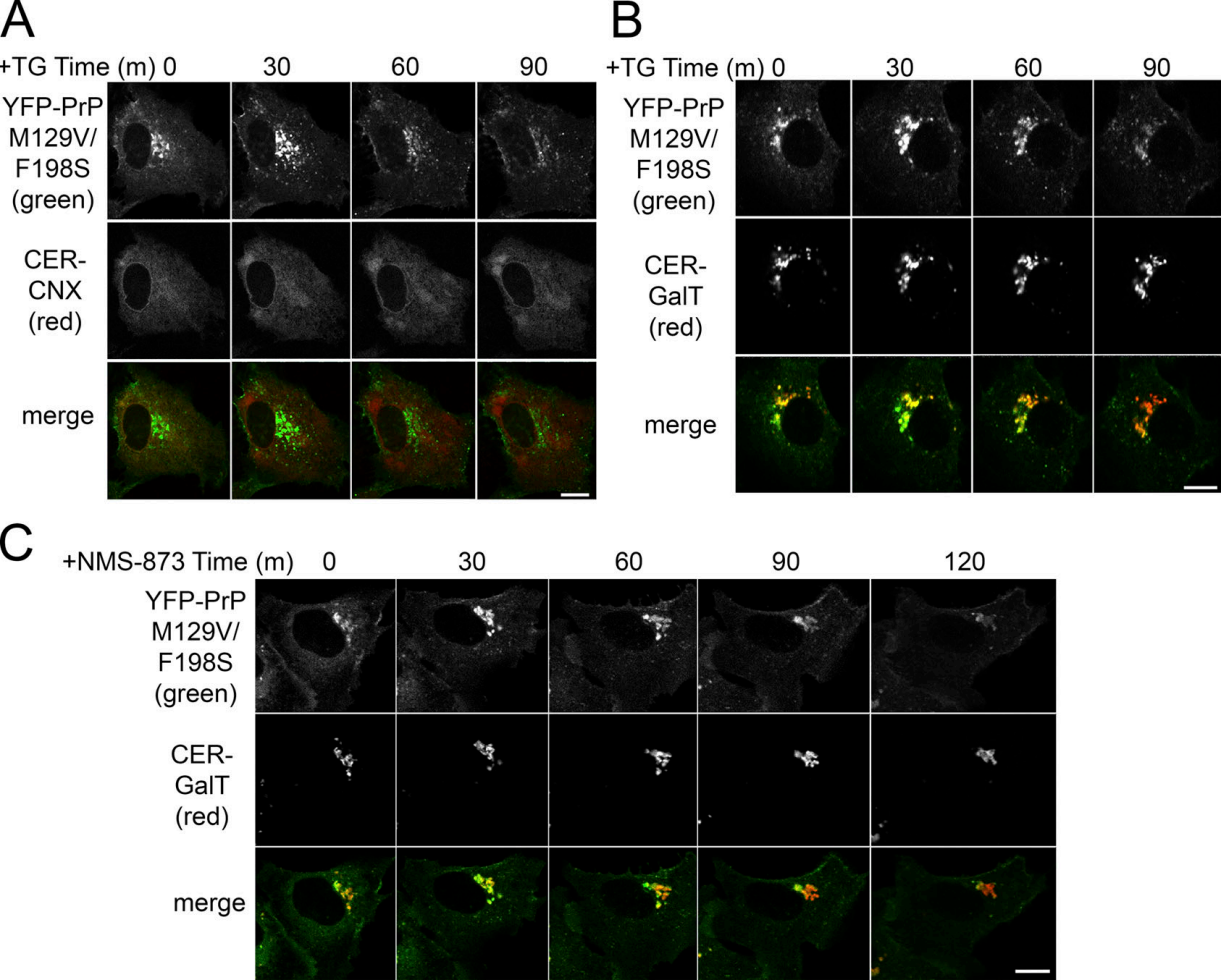
**Inhibiting ERAD increases the flux of disease mutant YFP-PrP M129V F198S through the RESET pathway. (A and B)** Time-lapse imaging of representative NRK cells transiently transfected with prion disease mutant, YFP-PrP M129V F198S, and (A) Cerulean (CER)-CNX or (B) CER-GalT after treatment with 1 μM thapsigargin. **(C)** Time-lapse imaging of a representative NRK cell transiently transfected with YFP-PrP M129V F198S and CER-GalT after treatment with 10 μM NMS-873. Scale bar, 10 μm.

We additionally tested familial Gerstmann–Straussler–Scheinker disease-associated mutant of PrP M129V F198S, which has partial populations localized to the ER, Golgi, and plasma membrane (Ivanova et al., 2001; Satpute-Krishnan et al., 2014). The ER-localized population of YFP-PrP M129V F198S is susceptible to undergoing RESET as revealed through the application of thapsigargin (Satpute-Krishnan et al., 2014; Fig. 3, A and B). We demonstrated that treatment with NMS-873 also induced the release of the ER population of YFP-PrP M129V F198S for RESET (Fig. 3 C and Video 1).

**Video 1.** **Inhibiting ERAD increases the flux of disease mutant YFP-PrP M129V F198S through the RESET pathway.** Time-lapse imaging of representative NRK cell transiently transfected with prion disease mutant, YFP-PrP M129V F198S, and Golgi marker, CER-GalT. Images were collected every 10 min after treatment with 10 μM NMS-873. Scale bar, 10 μm.

By blocking the major degradation pathway of newly synthesized misfolded ER PQC substrates, these experiments (Figs. 2 and 3) revealed a correlation between the accumulation of newly synthesized PQC substrates and the flux of ER-export of misfolded GPI-APs through the RESET pathway. This is in line with the idea that ER PQC substrates compete for binding to ER-resident chaperone(s). Next, we attempted to pinpoint the key ER-resident chaperone(s) that retained misfolded GPI-APs in the ER.

### RESET substrate association with CNX is stabilized upon global protein translation inhibition

**Figure 4.**
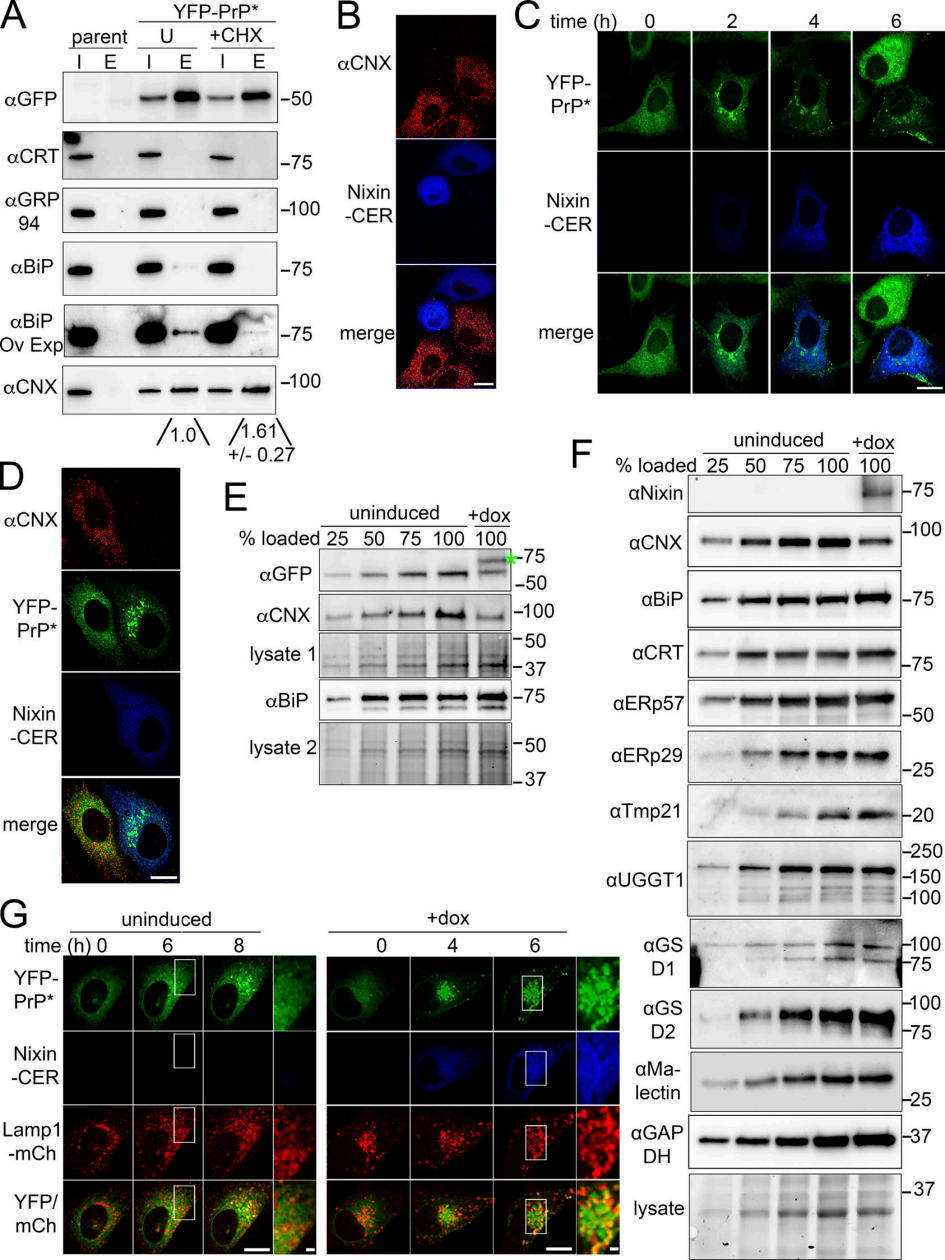
Calnexin is a key ER-retention factor for YFP-PrP*. (A) Western blots depicting GFP-column purifications from lysates of untransfected (parent) NRK cells or stably transfected YFP-PrP*NRK cells. Input “I” and eluate “E.” Cells were untreated (“U”) or treated with cycloheximide (“+CHX”) for 30 min, prior to lysis. For anti-calreticulin “αCRT” and anti-GRP94, no bands were detected in the eluate regardless of exposure time. For “αBiP OE,” the αBiP blot was overexposed to detect bands in the eluate. For anti-CNX, densitometry measurements showed the amount of CNX co-eluting with YFP-PrP* increased by 1.61 ± 0.27-fold after 30 m cycloheximide treatment. These results are representative of three independent experiments (*n* = 3, biological replicates) ± SD. Quantification of the relative pixel density of the CNX bands that copurified YFP-PrP* was made by creating a rectangular region of interest (ROI) that enclosed the larger “+CHX” eluate band, and then dragging the same ROI bounding box to enclose and measure the integrated density of the “+CHX” and untreated (“U”) CNX co-eluates. Integrated density values for the eluate CNX bands in the “+CHX” and “U” lanes were background subtracted and normalized against the “U” eluate band to obtain the relative pixel densities. **(B)** Immunofluorescence of CNX in NRK cells that were transfected with Nixin-Cerulean (CER). Field of view was selected to encompass Nixin-CER transfected and untransfected cells. **(C)** Time-lapse imaging of a representative YFP-PrP* NRK cell that was transiently transfected with Nixin-Cerulean. At the 6 h time point, an untransfected cell entered the field of view. **(D)** Immunofluorescence of CNX in YFP-PrP* NRK cells 6 h after transfection with Nixin-CER. Image includes transfected and untransfected cells, which provide an internal control. **(E and F)** Western blots of (E) YFP-PrP* NRK cells or (F) NRK cells that were stably transfected with doxycycline-inducible Nixin-CER either untreated (uninduced) or treated with doxycycline for 8 h (+dox). Bio-Rad Stain-free gel system was used to show total Lysate to confirm even loading. Samples were loaded into two gels. Total lysates labeled as “Lysate 1” corresponds with the anti-CNX and anti-GFP blot, to detect YFP-PrP* and Nixin-CER, and “Lysate 2” corresponds with anti-BiP/GRP78 blot. Lysates diluted to 25%, 50%, 75%, and undiluted from the uninduced cells were included as a reference. The blue asterisk marks the location of Nixin-CER. Glucosidase I, glucosidase II, and calreticulin were shortened to GSD1, GSD2, and CRT respectively. GAPDH was used as a loading control for the CNX, CRT, ERp57, and BiP blots. **(G)** Time-lapse imaging of bafilomycin A1 treated NRK cells that were stably expressing YFP-PrP* and Lamp1-mCherry (right) and transiently transfected with Nixin-CER (left). Lamp1-mCherry is a lysosomal marker. The inset is magnified on the right side of each image. Scale bars in the main figures are 10 μm, while scale bars in the insets are 1 μm. Source data are available for this figure: SourceData F4.

Since the SERCA pump inhibitor thapsigargin leads to the rapid depletion of ER calcium levels (Lytton et al., 1991) and immediate release of YFP-PrP* for ER-export (Satpute-Krishnan et al., 2014), we reasoned that the most likely candidates for ER-retention factors would include any of the major calcium-binding ER chaperones. If new protein synthesis induces the release of misfolded GPI-APs from ER-resident chaperones for ER-export, then, blocking translation with cycloheximide should stabilize the interaction between misfolded GPI-APs and those ER-resident chaperones. Therefore, to identify the chaperones involved in ER-retention of misfolded GPI-APs, we probed for interactions of YFP-PrP* with calcium-binding chaperones, CNX, calreticulin, GRP94, and BiP, in the absence and presence of cycloheximide (Fig. 4 A). We did not copurify detectable levels of calreticulin or GRP94 with YFP-PrP*, while long exposure times revealed a contingent of BiP coming down with YFP-PrP* that consistently decreased after 30 m of cycloheximide treatment. On the other hand, translation inhibition with cycloheximide clearly stabilized and increased the interaction between YFP-PrP* and CNX. Densitometry measurements of the western blots showed that the amount of CNX coeluting with YFP-PrP* increased by 1.61 ± 0.27-fold (*n* = 3) after 30 m of cycloheximide treatment. This cycloheximide-induced stabilization of YFP-PrP* and CNX implicated CNX’s involvement in ER-retention and release of RESET substrates.

### Induced degradation of CNX is concomitant with ER-export of RESET substrates

To further test whether CNX was indeed the key retention factor for RESET substrates, we attempted to deplete CNX. Unfortunately, our attempts to use CNX knockdown in NRK cells or a commercially available Hap1 CNX knockout cell line were not productive because CNX depletion appeared to prevent detectable expression of YFP-PrP*. The van der Goot group reported a similar issue in 2012 when they demonstrated that CNX knockdown suppresses the translocation and stable expression of GFP-PrP in transfected HeLa cells (Lakkaraju et al., 2012). We circumvented this problem instead by inducing the overexpression of a CNX-targeting E3 ubiquitin ligase to acutely downregulate CNX and monitoring the impact on YFP-PrP* trafficking in YFP-PrP* NRK cells.

Nixin/ZNRF4 is a RING finger E3 ubiquitin ligase that ubiquitinates CNX and triggers its rapid degradation in HeLa and HEK293T cells (Neutzner et al., 2011). We first confirmed by immunofluorescence that CNX was depleted in NRK cells that were transiently transfected with a Cerulean-tagged version of Nixin/ZNRF4 (Fig. 4 B). Next, we performed time-lapse imaging of YFP-PrP* NRK cells to reveal that rapid release of YFP-PrP* out of the ER was coincident with a new expression of Nixin-Cerulean (Fig. 4 C), and we verified by immunofluorescence that ER-export of YFP-PrP* was associated with CNX-depletion in cells expressing Nixin-Cerulean (Fig. 4 D).

To test whether Nixin-Cerulean expression specifically impacts CNX and not other major ER-resident chaperones, such as BiP or calreticulin, we created a YFP-PrP* NRK cell-line that was stably cotransfected with a Tet-inducible Nixin-Cerulean for acute induction of Nixin-Cerulean expression. YFP-PrP* NRK cells were cotransfected with the Tet-inducible Nixin-Cerulean and enriched for an ∼50% population of stable transfectants. After 8 h of doxycycline induction, Nixin-Cerulean expression resulted in ∼50% decrease of CNX and YFP-PrP* levels as compared with uninduced cells but did not cause a decrease in BiP or calreticulin levels (Fig. 4, E and F).

Next, we addressed the possibility that Nixin was able to directly or indirectly induce the degradation of other known CNX-associated factors that could play a role in YFP-PrP* retention. In cells expressing doxycycline-inducible Nixin-Cerulean, we probed for the known calnexin-cycle members (UGGT1, glucosidase I, glucosidase II, malectin), two well-characterized CNX-associated PDIs (ERp29 and ERp57), and the requisite RESET factor Tmp21. Again, while CNX expression levels dropped in response to Nixin-Cerulean induction, we did not detect an obvious increase or decrease in the expression of these associated proteins (Fig. 4 F). Although our candidate approach did not reveal other potential ER-retention factors that were degraded upon Nixin induction, additional unknown ER-retention factors that function in conjunction with CNX could be revealed by a complete proteomic comparison between Nixin-overexpressing cells and control cells, which is outside the scope of this study. Regardless of whether CNX acts alone or with an associated ER-retention factor, our data implicates CNX as a key retention factor for RESET substrates.

Finally, we tested whether YFP-PrP*’s itinerary culminated in lysosomes after Nixin-Cerulean-induced release from the ER, which, as previously reported, occurs upon ER-release during steady-state conditions (Satpute-Krishnan et al., 2014). We cotransfected YFP-PrP* NRK cells with Dox-inducible Nixin-Cerulean and the lysosomal marker Lamp1-mCherry. Treatment with bafilomycin A1 to block lysosomal degradation revealed that a small but detectable fraction of YFP-PrP* accumulated in lysosomes over 6 h under steady-state conditions, as expected, and that the release of YFP-PrP* to lysosomes was strongly enhanced by doxycycline-induced Nixin-Cerulean expression (Fig. 4 G). Thus, utilizing Nixin/ZNRF4 to degrade CNX released YFP-PrP* for ER-export but did not impact its ultimate fate: lysosomal delivery. The correlation between the rapid depletion of CNX and the rapid release of YFP-PrP* for RESET supports the idea that CNX is a key ER-retention factor involved in regulating the flux of RESET substrates.

### Increased production CNX-binding substrates through deoxynojirimycin treatment enhances the flux of RESET substrate trafficking from the ER to the Golgi

Almost all nascent secretory pathway proteins are modified in the ER with an N-linked glycosylation precursor that includes three terminal glucose molecules that are processed by glucosidases I and II down to a single glucose for recognition by the lectin chaperones, CNX and calreticulin (Hammond et al., 1994; Hebert et al., 1995). 1-Deoxynojirimycin (DNJ) is an inhibitor of both glucosidases I and II that trim the terminal glucose molecules on the N-linked glycans (Hebert et al., 1995; Saunier et al., 1982). Therefore, treatment with DNJ would be expected to trap the newly added N-linked glycans in the triglucosylated state. However, in the context of primary and secondary cell cultures derived from rat, DNJ was reported to induce a buildup of monoglucosylated versions of N-linked glycoproteins (Gross et al., 1983, 1986; Romero et al., 1985). Accordingly, we reasoned that if DNJ allows for the buildup of newly synthesized CNX-binding glycoproteins in our rat-derived YFP-PrP* NRK cell line, then they may compete with YFP-PrP* for association with CNX and thus induce the release of YFP-PrP* from CNX for ER-export.

**Figure 5.**
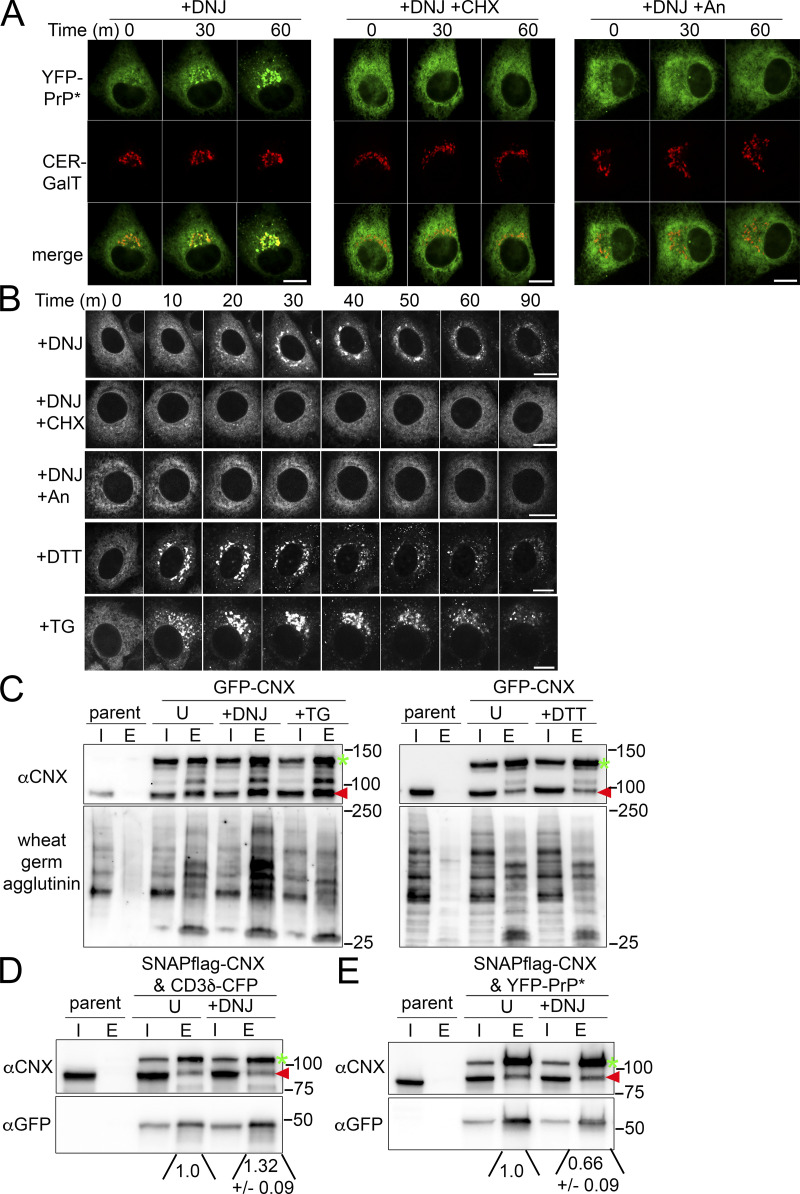
Deoxynojirimycin-treatment enhances the flux of YFP-PrP* through the RESET pathway, the binding of glycoproteins to CNX, and the release of YFP-PrP* from CNX. (A) Time-lapse imaging of representative YFP-PrP* and Cerulean (CER)-GalT NRK cells after treatment with 2.5 mM deoxynojirimycin alone (DNJ) or with 50 μg/ml cycloheximide (DNJ+CHX), or with 0.5 μM anisomycin (DNJ+An). CER-GalT is a Golgi marker. **(B)** Time-lapse imaging of representative YFP-PrP* NRK cells that were treated with 2.5 mM DNJ, DNJ+CHX, DNJ, DNJ+An, 0.5 mM dithiothreitol (DTT), or 1 μM thapsigargin (TG). **(C)** Representative western blots depicting GFP-column purifications from N2a cell lysates of untransfected (parent) cells or GFP-CNX-transfected cells 48 h after transient transfection. Blots were probed with anti-CNX (αCNX) antibody to detect GFP-CNX (green arrow) or endogenous CNX (red arrowhead) that co-eluted with GFP-CNX or probed with wheat germ agglutinin to detect general glycosylated proteins that co-eluted with GFP-CNX. Input “I” and eluate “E.” Cells were untreated (“U”) or treated with DNJ, TG, and DTT for 30 min, as indicated, prior to lysis. The experiment was performed in triplicate with similar results. **(D and E)** Representative western blots depicting flag-column purifications from N2a cell lysates of untransfected (parent) cells or transfected cells ∼72 h after transient transfection with (D) SNAPflag-CNX and CD3δ-CFP or (E) SNAPflag-CNX and YFP-PrP*. Blots were probed with anti-CNX (αCNX) antibody to detect SNAPflag-CNX (green arrow) or endogenous CNX (red arrowhead) that coeluted with SNAPflag-CNX and with anti-GFP antibody to probe for (D) CD3δ-CFP or (E) YFP-PrP* that co-eluted with SNAPflag-CNX. Input “I” and eluate “E.” Cells were untreated “U” or treated with DNJ for 15 m, as indicated, prior to lysis. Experiments were performed in triplicate. Integrated densitometry measurements were made of the αGFP bands. Quantification of the relative pixel density of the αGFP bands that co-purified with SNAPflag-CNX was made by first creating a rectangular bounding box or region of interest (ROI) that enclosed the larger eluate band. For D, the larger αGFP band was in the +DNJ eluate lane. For E, the larger αGFP band was in the untreated “U” eluate lane. Next, for D and E respectively, the ROI that enclosed the larger αGFP eluate band was dragged to enclose and measure the integrated density of αGFP eluate band untreated “U” and "+DNJ" co-eluates, and integrated densities within the bounding box were measured. Finally, the eluate αGFP bands in the "U" and "+DNJ" lanes were background subtracted and normalized against the “U” eluate band to obtain the relative pixel densities. These results are representative of three independent experiments (*n* = 3, biological replicates) ± SD. Source data are available for this figure: SourceData F5.

**Figure S3.**
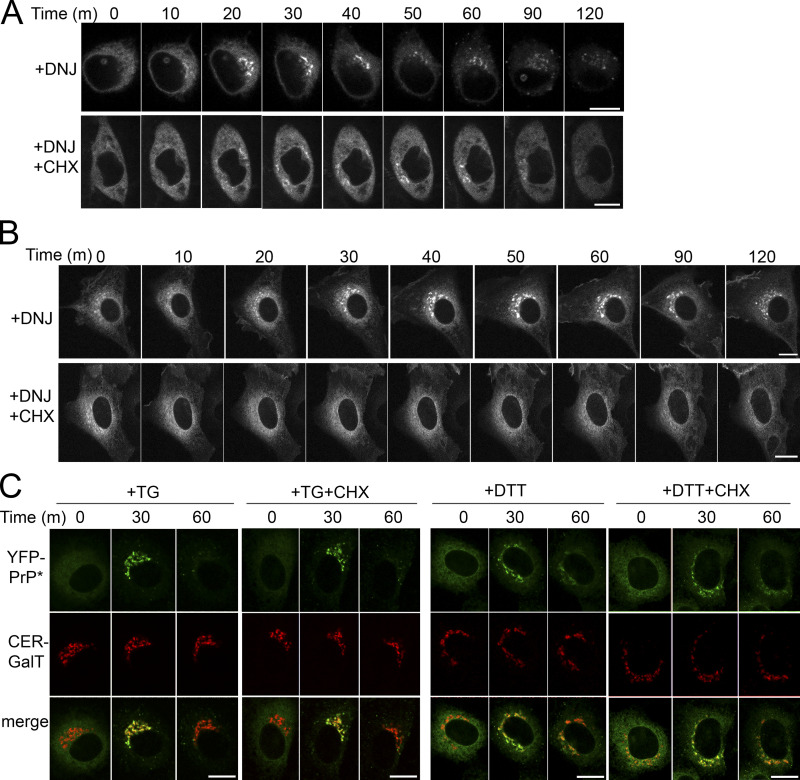
**Deoxynojirimycin, but not thapsigargin or dithiothreitol, induces RESET of misfolded GPI-APs through a mechanism that requires constitutive protein translation. (A)** Time-lapse imaging of representative N2a cell 48 h after transient transfection with YFP-PrP* and treated with 2.5 mM 1-deoxynojirimycin (DNJ) alone, or DNJ and 50 μg/ml cycloheximide (DNJ+CHX). **(B)** Time-lapse imaging of representative NRK cells that were stably expressing GFP-CD59 C94S after treatment with DNJ alone, or DNJ+CHX. **(C)** Time-lapse imaging of representative YFP-PrP* NRK cells after treatment with 1 μM thapsigargin alone (TG) or with TG+CHX, or 5 mM dithiothreitol alone (DTT) or with CHX (DTT+CHX). Scale bar, 10 μm.

Time-lapse imaging revealed that incubating YFP-PrP* NRK cells with 2.5 mM DNJ treatment triggered the release of a fraction of the YFP-PrP* molecules to the Golgi within 30 m (Fig. 5, A and B). Critically, including cycloheximide with DNJ counteracted the DNJ-induced release of YFP-PrP*, suggesting a role for new protein synthesis (Fig. 5, A and B). Likewise, coincubation of an alternate protein translation inhibitor, anisomycin (Garreau de Loubresse et al., 2014), with DNJ also prevented DNJ-induced RESET of YFP-PrP* (Fig. 5, A and B). We obtained similar results with YFP-PrP*-expressing N2a cells or eGFP-CD59 C94S NRK cells treated with DNJ or DNJ with cycloheximide (Fig. S3, A and B). Notably, comparison between time-lapses of YFP-PrP* NRK cells revealed that DNJ induces RESET of YFP-PrP* less efficiently than the chemical ER stressors, thapsigargin or dithiothreitol (Fig. 5, A and B; and Fig. S3 C), which clear nearly all of the YFP-PrP* out of the ER into the Golgi within 30 m as previously quantified (Satpute-Krishnan et al., 2014). Additionally, cycloheximide did not inhibit thapsigargin or dithiothreitol-induced RESET (Fig. 1 H and Fig. S3 C), demonstrating that these acute ER stressors release YFP-PrP* from the ER through a different mechanism from DNJ, a mechanism that is not dependent on new CNX substrate production.

Since CNX appears to play a critical role in the retention of RESET substrates (Fig. 4), we hypothesized that DNJ may induce the release of YFP-PrP* from the ER by causing a buildup of CNX-binding glycoproteins that may potentially compete with RESET substrates for association with CNX. To determine if DNJ induced a buildup of CNX-binding substrates, we column-purified exogenously expressed GFP-CNX from untreated or DNJ-treated N2a cells, performed SDS-PAGE and western blot, and probed the membrane with the lectin, wheat germ agglutinin (WGA). Since WGA binds to *N*-acetylglucosamine (GlcNAc) and sialylated glycans found in a variety of N-linked and O-linked carbohydrate structures (Bhavanandan and Katlic, 1979; Cummings et al., 2022; Nagata and Burger, 1974; Trinidad et al., 2013), we used it as a general probe for glycoproteins, including those modified with N-linked glycans in the ER. Treatment with DNJ caused a clear increase in cellular glycoproteins that coeluted with GFP-CNX, while thapsigargin and dithiothreitol did not (Fig. 5 C). Thus, DNJ increased overall substrates bound to CNX while triggering the release of YFP-PrP* for RESET.

Based on our competition model, we predicted that although DNJ induces an overall increase in CNX-binding substrates, DNJ would specifically reduce the binding of RESET substrates to CNX. We compared the effect of DNJ treatment on the association between CNX and either our model RESET substrate, YFP-PrP*, or an established ERAD substrate CD3δ that is well known to interact with CNX (David et al., 1993; Kearse, 1998; van Leeuwen and Kearse, 1996). We initially attempted to perform experiments testing for CNX association in YFP-PrP*-expressing cells that were cotransfected with CD3δ-CFP. However, while co-transfection of CD3δ-CFP in YFP-PrP* NRK or N2a cells initially caused RESET of YFP-PrP*, described below, cells that co-expressed both PQC substrates eventually underwent apoptosis within 24 h (not shown). We were thus required to study interactions between CNX and PrP* or CNX and CD3δ separately in live cells.

Since a detectable fraction of YFP-PrP* was released from the ER within 10–20 m of DNJ treatment (Fig. 5 B and Fig. S3 A), anti-flag column purification of SNAPflag-CNX from cells coexpressing SNAPflag-CNX and either CD3δ-CFP or YFP-PrP* were performed 15 m after DNJ treatment. Blots were probed with anti-CNX antibody to detect SNAPflag-CNX or endogenous CNX and anti-GFP antibody to detect CD3δ-CFP or YFP-PrP* that copurified with the SNAPflag-CNX. The amount of CD3δ-CFP that coeluted with SNAPflag-CNX increased by 1.32 (±0.09 SD)-fold (*n* = 3) in DNJ-treated conditions as compared with untreated conditions (Fig. 5 D). By contrast, the amount of YFP-PrP* that coeluted with SNAPflag-CNX after DNJ treatment decreased to 0.66 (±0.09 SD)-fold (*n* = 3) as that of untreated conditions (Fig. 5 E). Incidentally, DNJ treatment did not noticeably impact the oligomerization of CNX; with or without DNJ treatment, the exogenously expressed, tagged CNX co-eluted with similar amounts of endogenous CNX (Fig. 5, C–E). The inverse correlation between CNX-bound RESET substrate, YFP-PrP*, and other N-linked glycoproteins (i.e., lectin-binding general pool or CD3δ-CFP) implicates competitive binding to CNX as a mechanism that induces the release of RESET substrates for ER-export.

### Deoxynojirimycin induces clearance of YFP-PrP* from the ER via the RESET pathway

**Figure 6.**
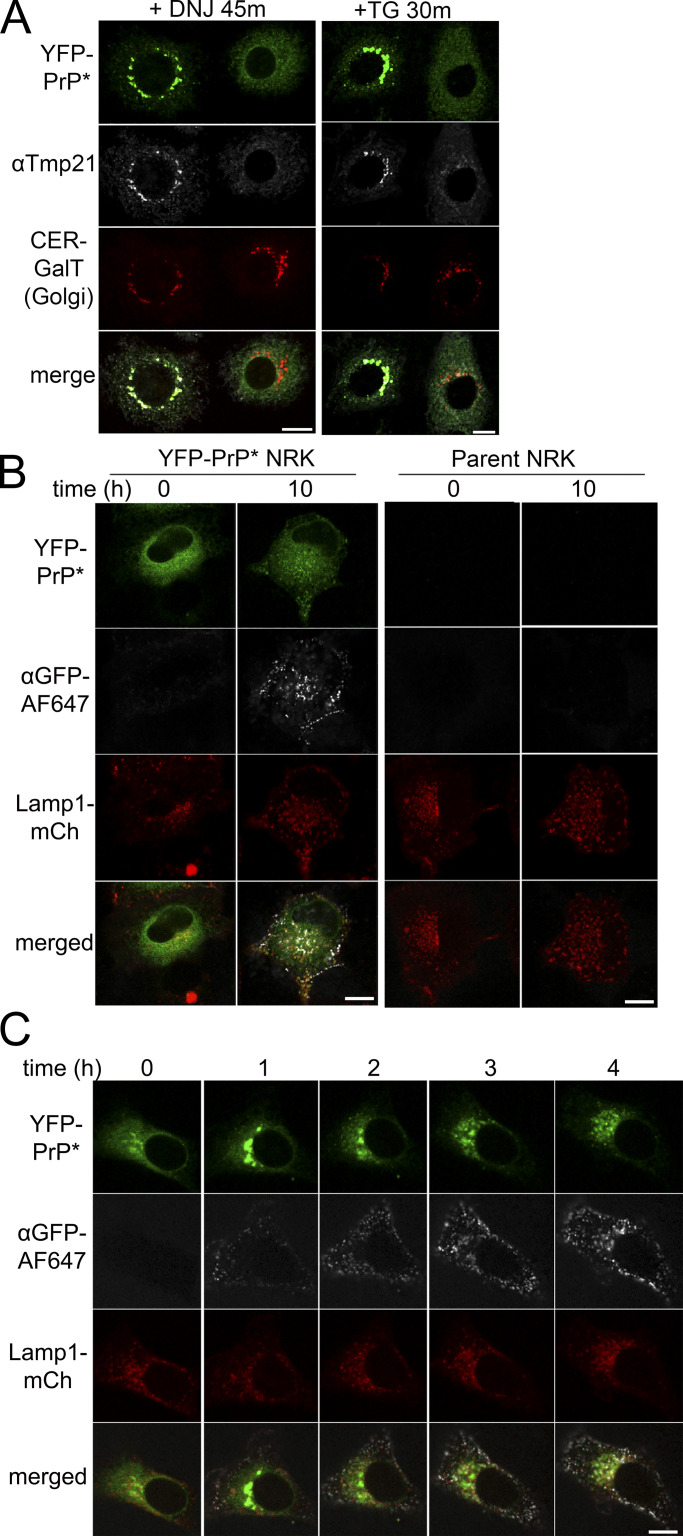
Deoxynojirimycin-enhances release of YFP-PrP* for ER-export by a pathway that is dependent on Tmp21 and results in transient exposure on the cell surface. (A) Images of representative YFP-PrP* CER-GalT NRK cells that were subjected to Tmp21 siRNA knockdown treatment followed by treatment with 2.5 mM deoxynojirimycin (“+DNJ”) for 45 min or 0.1 µM thapsigargin (“+TG”) for 30 min before fixation and immunofluorescence against Tmp21. Each panel shows two adjacent cells in the same field of view where one cell was fully depleted for Tmp21 (cell on right) and the other cell was not (cell on left). For cells treated with DNJ for 45 min, quantification of 107 randomly imaged Tmp21-depleted cells revealed that YFP-PrP* is localized to the ER in 100% of the Tmp21-depleted cells. For cells treated with TG for 30 min, quantification of 53 randomly imaged Tmp21-depleted cells revealed that YFP-PrP* is localized to the ER in 100% of the Tmp21-depleted cells, confirming a finding previously described (Satpute-Krishnan et al., 2014). **(B)** Time-lapse imaging of an NRK cell stably expressing both YFP-PrP* and lysosome-marker Lamp1-mCherry, labeled “YFP-PrP* NRK,” or Lamp1-mCherry alone, labeled “Parent NRK.” Cells were incubated with 250 nM bafilomycin A1 and AlexaFluor (TM) 647-conjugated anti-GFP antibody (αGFP-AF647). **(C)** Time-lapse imaging of an NRK cell stably expressing both YFP-PrP* and Lamp1-mCherry, incubated with 2.5 mM 1-deoxynojirimycin, 250 nM bafilomycin A1, and αGFP-AF647. Identical imaging parameters were applied for all cells imaged for B and C. Scale bar, 10 μm.

To confirm that DNJ induces ER-export of YFP-PrP* by the RESET pathway as previously described in thapsigargin-treated cells (Satpute-Krishnan et al., 2014), we first tested whether Tmp21 knock-down inhibited ER-to-Golgi transport of YFP-PrP*. We imaged randomly selected Tmp21-depleted YFP-PrP* NRK cells that were either treated with DNJ for 45 m (representative cell shown in Fig. 6 A, left panel [*n* = 107]) or thapsigarin for 30 m (representative cell shown in Fig. 6 A, right panel [*n* = 53]) and found that YFP-PrP* remained in the ER in 100% of the DNJ-treated or thapsigargin-treated Tmp21-depleted cells. On the other hand, it trafficked to the Golgi in cells expressing endogenous Tmp21. Thus, DNJ-induced ER-export of YFP-PrP* is absolutely dependent on Tmp21.

Next, we tested whether YFP-PrP* released from the ER during DNJ treatment accesses the cell surface where it could bind and internalize Alexa Fluor 647-conjugated anti-GFP antibodies en route to lysosomes, as previously demonstrated during steady-state or thapsigargin-induced ER stress conditions (Satpute-Krishnan et al., 2014). Under steady-state conditions, in untreated YFP-PrP* NRK cells, internalized antibody was first detected within 30 m and accumulated over 10 h, but was not detected under identical conditions in parental untransfected NRK cells (Fig. 6 B and Video 2), establishing the specificity for the uptake of Alexa Fluor 647-conjugated anti-GFP antibodies. YFP-PrP* released from the ER during DNJ treatment was able to internalize the antibody (Fig. 6 C and Video 3).

**Video 2.** Steady-state trafficking of YFP-PrP* to the cell surface results in antibody uptake. Time-lapse imaging of two representative NRK cells. The cell shown on the left was stably expressing both YFP-PrP* and lysosome-marker Lamp1-mCherry. The cell shown on the right was stably expressing only the lysosome marker Lamp1-mCherry and thus was labeled “untransfected” for YFP channel. Cells were incubated with 250 nM bafilomycin A1 and AlexaFluor (TM) 647-conjugated anti-GFP antibody (αGFP-AF647) at *t* = 0. Images were collected every 30 min for 10 h. Scale bar, 10 μm.

**Video 3.** Deoxynojirimycin-induced release of YFP-PrP* for ER-export results in antibody uptake. Time-lapse imaging of YFP-PrP* NRK cell stably expressing Lamp1-mCherry taken after the addition of 2.5 mM 1-deoxynojirimycin, 250 nM bafilomycin A1, and anti-GFP-AF647. Images were collected every 10 m for 6 h. Scale bar, 10 μm.

Thus, these observations confirm that DNJ-induced ER-clearance of YFP-PrP* occurs via the RESET pathway as defined by criteria first described (Satpute-Krishnan et al., 2014): (1) dependence on the requisite RESET factor Tmp21 and (2) subsequent release to the cell surface followed by endocytosis and lysosomal degradation.

### Induction of CNX folding substrates triggers the release of RESET substrates for ER-export

N-linked glycoproteins are acutely upregulated during a variety of conditions such as tumorigenesis (Tax et al., 2019), inflammatory conditions (Chen et al., 2021), or viral infections (Molinari, 2002). Specific examples include Thy1 (Jósvay et al., 2014; Lee et al., 1998), IL-6 (Kaur et al., 2020; Narazaki and Kishimoto, 2018), HHV-8 vIL6 (Nicholas, 2007; Sakakibara and Tosato, 2011), and SARS-CoV-2 (Santopolo et al., 2021). Given that nascent secretory pathway substrates typically interact with CNX during their folding process (Hammond et al., 1994; Hebert et al., 1995), we hypothesized that acute overexpression of diverse secretory pathway substrates may induce RESET of YFP-PrP*.

**Figure 7.**
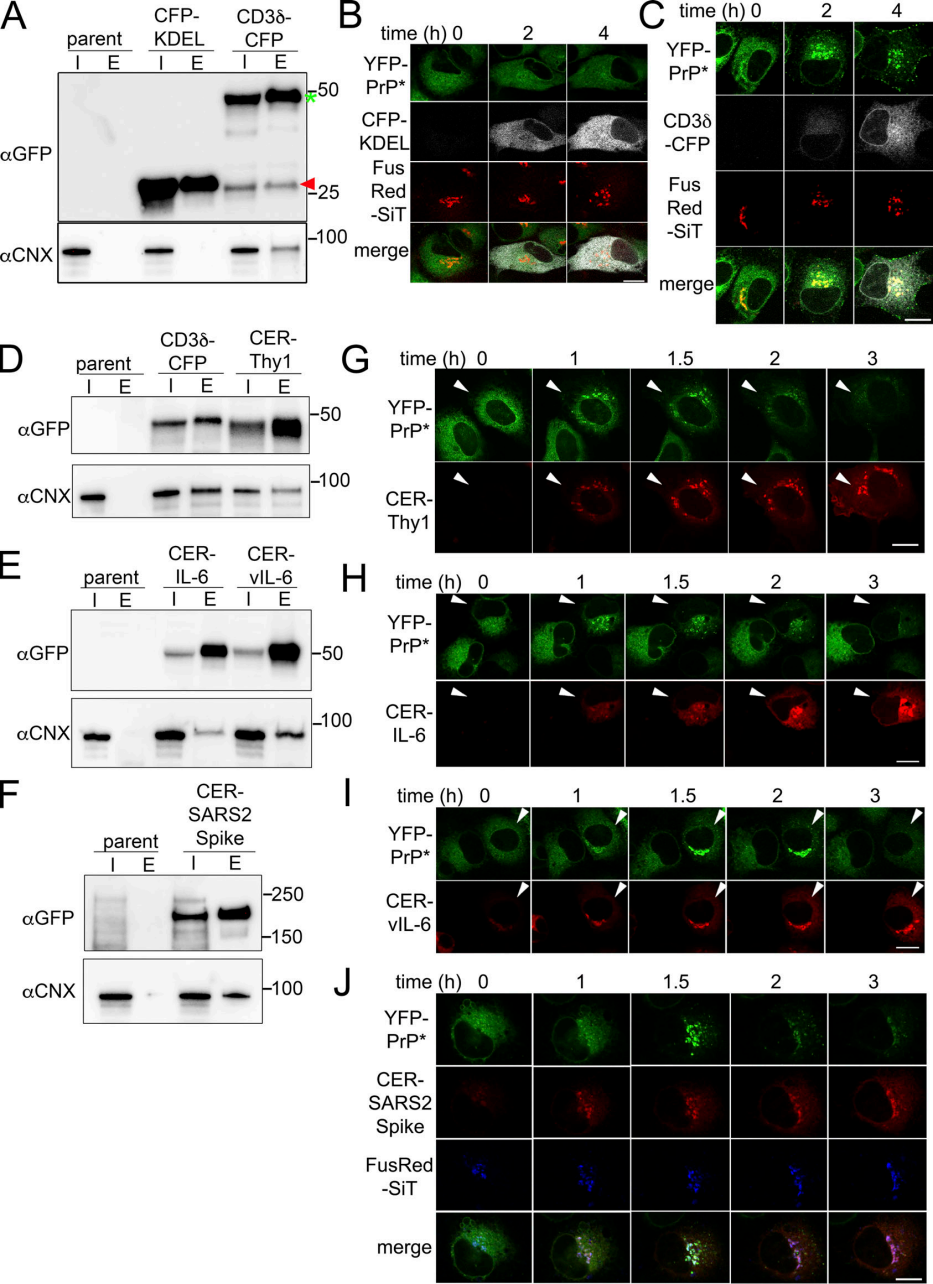
New expression of CNX-binding N-linked glycoproteins triggers RESET of PrP*. (A) Western blots depicting GFP column purifications from parental, untransfected N2a cells (parent), or N2a cells 12 h after transient transfection with ER-targeted CFP-KDEL or CD3δ-CFP. Blots were probed with anti-GFP antibodies to detect CFP-KDEL or CD3δ-CFP and with anti-CNX antibody to detect endogenous CNX. Blue asterisk and red arrowhead placed next to the GFP-blot align with full-length CD3δ-CFP and CFP-KDEL, respectively. Input “I,” eluate “E.” **(B and C)** Time-lapse imaging of representative NRK cells stably expressing YFP-PrP* and the Golgi marker, FusionRed-SiT (FusRed-SiT), and transiently transfected with (B) ER-targeted CFP (CFP-KDEL) or (C) CD3δ-CFP. Image collection was started 2 h after transient transfection. Scale bar, 10 μm. **(D–F)** Western blots depicting GFP column purifications from parental, untransfected N2a cells (parent) or N2a cells 12 h after transiently transfecting cells with (D) CD3δ-CFP or Cerulean-Thy1, (E) Cerulean-IL-6 or Cerulean-vIL-6, and (F) Cerulean-SARS CoV-2 Spike Glycoprotein “Cer-SARS2 Spike,” and probed with anti-GFP antibody or anti-CNX antibody. Input “I” and eluate “E.” **(G–J)** Time-lapse imaging of YFP-PrP* NRK cells that were transiently transfected with Cerulean-tagged N-linked glycoproteins that were reported to be upregulated during inflammatory conditions, including (G) Thy1, (H) IL-6, and during a viral infection (I) vIL-6 and (J) SARS2 Spike. For G–I, the fields of view include one cell that was transfected with the Cerulean-tagged N-linked glycoprotein and one cell that remained untransfected. The arrowheads point to cells which were newly expressing the transfected N-linked glycoproteins. For J, cells stably expressed Golgi marker, FusRed-SiT. For G–J, image collection was started 2 h after addition of transfection reagent and plasmids. Scale bar is 10 um. Source data are available for this figure: SourceData F7.

**Figure S4.**
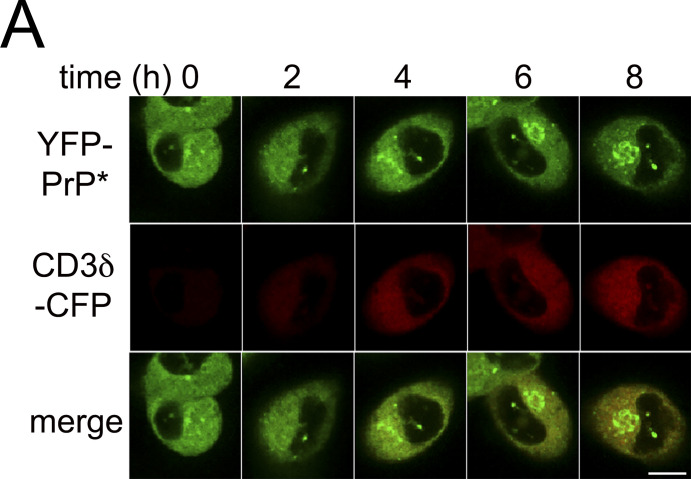
New expression of CD3δ triggers RESET of PrP*. Time-lapse imaging of a representative N2a cell 48 h after transfection with YFP-PrP*, followed by a second round of transient transfection with CD3δ-CFP. Image collection was started 2 h after transfection with CD3δ-CFP. As with all time-lapse imaging, the same cell was imaged over time. Typical of N2a cells, this cell is mobile and rolls around several times over the course of this time-lapse sequence. Scale bar, 10 μm.

As a first step toward testing whether the induction of specific CNX folding substrates influences the flux of traffic through the RESET pathway, we used the established CNX substrate and N-linked glycoprotein CD3δ-CFP as a positive control. We used the ER-targeted CFP molecule, ER-mCFP-KDEL (“CFP-KDEL”; Snapp et al., 2006), as a negative control. We verified by GFP-column purification that newly expressed CD3δ-CFP associates with CNX, while CFP-KDEL does not (Fig. 7 A). We prepared YFP-PrP* NRK cells that stably expressed the Golgi marker, FusionRed-SiT-15, and transiently transfected them with CD3δ-CFP or CFP-KDEL. Time-lapse imaging revealed that new expression of CD3δ-CFP caused a dramatic shift of YFP-PrP* out of the ER to the Golgi for the RESET pathway, while CFP-KDEL did not alter the localization of ER-retained YFP-PrP* (Fig. 7, B and C). New CD3δ-CFP expression also triggered the release of YFP-PrP* for RESET in N2a cells (Fig. S4).

Second, we tested diverse, naturally upregulated CNX-binding substrates for their ability to induce RESET. Thy1 (Satpute-Krishnan et al., 2014), HHV-8 vIL-6 (Chen et al., 2009), and the close relative of SARS-CoV-2 spike glycoprotein, SARS-CoV spike glycoprotein (Fukushi et al., 2012), have each been predicted or reported to be CNX-folding substrates during synthesis. We confirmed that newly expressed Cerulean-tagged Thy1, IL-6, vIL-6, and SARS-CoV-2 spike glycoprotein each associate with CNX during new expression (Fig. 7, D–F) and trigger RESET of YFP-PrP* (Fig. 7, G–J).

**Figure 8.**
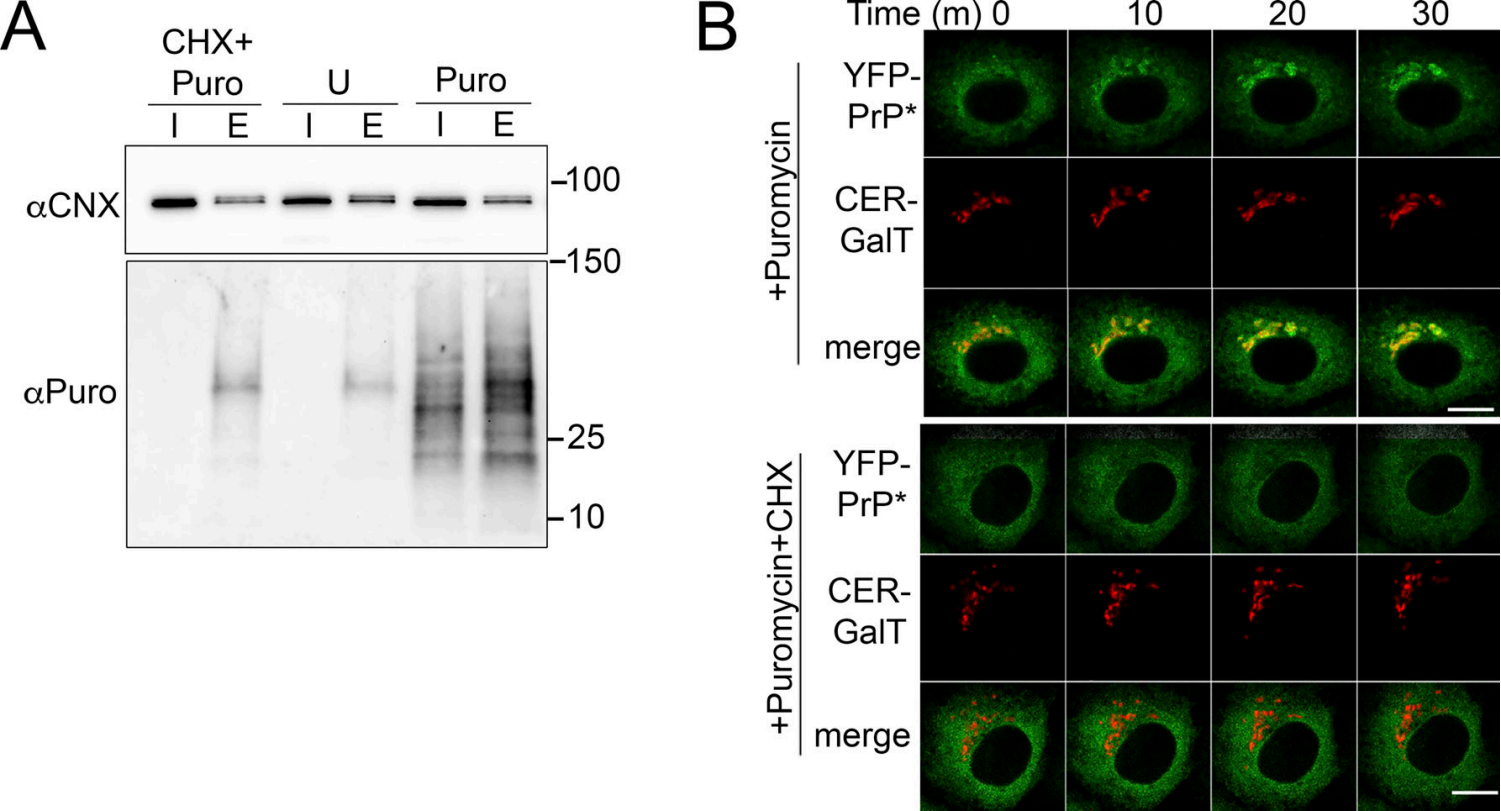
Puromycin treatment of cells produces puromycinylated CNX-associated products and induces RESET of YFP-PrP* by a mechanism that is dependent on new protein synthesis. (A) Western blots depicting column purifications of endogenous CNX from lysates of NRK cells treated with 50 μg/ml cycloheximide and 2.50 µg/ml of puromycin (“CHX+Puro”), untreated (“U”), or treated with puromycin with 2.50 µg/ml of puromycin alone (“Puro”). Blots were probed with anti-CNX (αCNX) antibody to detect endogenous CNX and anti-puromycin antibody to detect puromycinylated proteins products that co-eluted with CNX. Input “I” and eluate “E.” **(B)** Time-lapse imaging of representative YFP-PrP* and Cerulean (CER)-GalT NRK cells after treatment with 2.50 µg/ml of puromycin alone (“+Puromycin”) or together with 50 μg/ml cycloheximide (“+Puromycin+CHX”), CER-GalT is a Golgi marker. Source data are available for this figure: SourceData F8.

Third, we tested puromycin, which acts through a different mechanism from DNJ to acutely produce a broad spectrum of nascent, misfolded products, including CNX-binding substrates. Puromycin is a structural analog of aminoacyl tRNAs that gets incorporated into newly forming polypeptide chains causing the ribosome to prematurely release truncated C-terminally puromycinylated polypeptide chains (Nathans, 1964). Although puromycin indiscriminately blocks the translation of proteins across the cell, a detectable fraction of the puromycinylated proteins enter the secretory pathway (Schmidt et al., 2009), and importantly, have been shown to associate with CNX (Zhang et al., 1997). We replicated experiments confirming the interaction between CNX and puromycinylated proteins within 30 min of puromycin treatment by column-purifying endogenous CNX and probing for bound puromycinylated proteins. Coincubation with cycloheximide blocked the production of puromycinylated proteins and by extension precluded CNX from copurifying with puromycinylated proteins (Fig. 8 A). This demonstrated that the production of puromycinylated CNX substrates is dependent on new protein synthesis. Like other treatments that result in the accumulation of PQC substrates in the ER, such as MG132, NMS-873, and DNJ, puromycin treatment induces the release of YFP-PrP* for RESET, while cycloheximide prevents puromycin-induced RESET (Fig. 8 B).

Taken together, this study demonstrates that the induction of CNX-binding substrates through diverse mechanisms triggers the release of misfolded GPI-APs from CNX and increases their flux through the RESET pathway (Figs. 2, 3, 5, 6, 7, 8, S2 B, S3, and S4). On the other hand, blocking the production of new PQC substrates with translation inhibitors stabilizes the association of misfolded GPI-APs with CNX (Fig. 4 A) and inhibits their release for RESET (Figs. 1, 2, 5, 8, S2 B, and S3). One likely explanation for these observations is that newly synthesized secretory pathway proteins compete with misfolded GPI-APs for binding to CNX.

### Increased binding of substrates to CNX correlates with the release of RESET substrates from CNX

**Figure 9.**
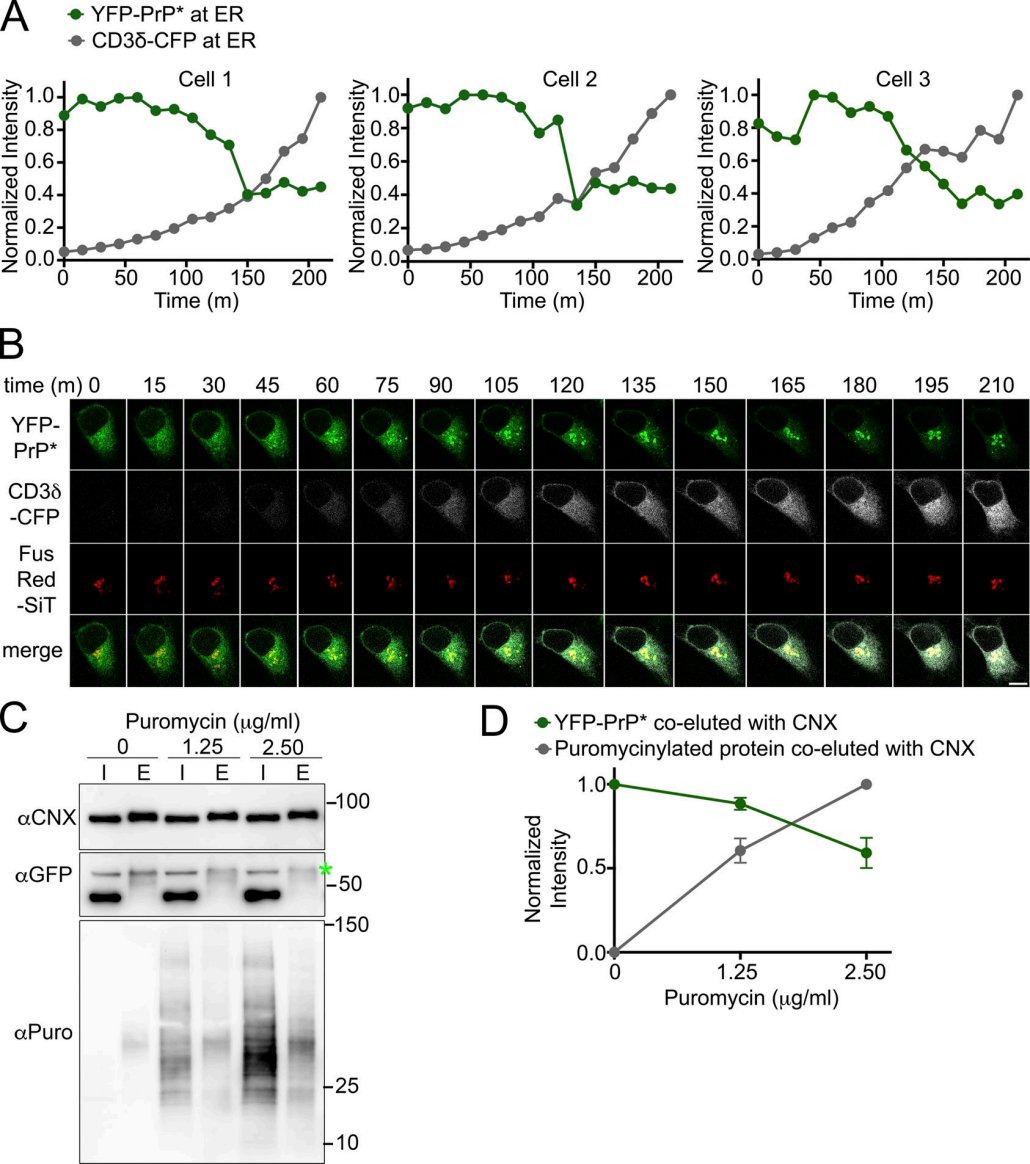
The levels of CNX-binding substrates are inversely correlated with the levels of ER-localized or CNX-bound YFP-PrP*. (A) Time traces of normalized total intensity of YFP-PrP* (green) and CD3δ-CFP (gray) in the ER of three individual cells (Cell 1–3) that were imaged every 15 min upon the induction of new CD3δ-CFP expression. Details for how the time traces were generated are provided in the Materials and methods section. **(B)** Montage of time-lapse for Cell 1 depicted in A showing YFP-PrP* (green), CD3δ-CFP (grayscale), and the Golgi marker, FusionRed-SiT (FusRed-SiT) (red). **(C)** Western blots depicting column purifications of endogenous CNX from lysates of YFP-PrP* NRK cells that were treated with 0, 1.25, or 2.50 µg/ml of puromycin for 30 min. Input “I” and eluate “E.” 24 h prior to column purification (described in Materials and methods), equal numbers of cells were seeded into 10-cm dishes and cells were processed identically, with the exception of the 30 m puromycin treatments. Blots were probed with anti-CNX “αCNX” antibody to detect endogenous CNX, anti-GFP “αGFP” antibody to detect YFP-PrP*, and anti-puromycin “αPuro” antibody to detect puromycinylated proteins products that co-eluted with CNX. **(D)** Plots of the relative pixel densities representing YFP-PrP* and puromycinylated-proteins that co-eluted with endogenous CNX. To quantify the relative amounts of the YFP-PrP* or puromycinylated-proteins that co-purified with CNX, we performed the following steps. The integrated densities of the YFP-PrP* or puromycinylated co-eluates for each of the 0, 1.25 and 2.50 µg/ml puromycin-treated samples were measured within a bounding box or region of interest (ROI) sized to enclose the YFP-PrP* band in the 0 ug/ml puromycin eluate “E” lane or all of the bands spanning the 2.5 µg/ml puromycin eluate “E” lane, respectively, and background subtracted. To address the variation in CNX pulled down from the different samples, integrated densities of co-eluted YFP-PrP* or puromycinylated-products were normalized against the integrated densities of the corresponding CNX bands to produce relative amounts of PrP or puromycinylated-proteins that were bound to CNX. Finally, to facilitate evaluation of the proportional changes between samples, values were further normalized such that CNX-bound YFP-PrP* in untreated “0” cells were normalized to 1, and CNX-bound puromycinylated proteins were normalized so that the entire range falls between 0 (for untreated sample) to 1 (for the 2.5 μg/ml puromycin-treated sample). The plotted relative integrated densities are representative of three independently performed experiments (*n* = 3, biological replicates) ± SD. The relative values of CNX-associated YFP-PrP* in 0, 1.25, and 2.50 µg/ml puromycin-treated cells were 1.00, 0.88 ±0.04 (SD), and 0.59 ±0.09 (SD), respectively. The relative values of total CNX-associated puromycinylated-proteins in 0, 1.25, and 2.50 µg/ml puromycin-treated cells were 0, 0.60 ±0.07, and 1, respectively. Source data are available for this figure: SourceData F9.

We hypothesized that the flux of traffic through the RESET pathway depends on competition between the general population of newly synthesized secretory pathway substrates and the pool of RESET substrates for association with CNX. If competition were a factor, then the levels of transiently transfected CNX substrates would inversely correlate with the levels of RESET substrates retained in the ERs of individual cells. Exogenously expressed CD3δ-CFP is a particularly useful model substrate for this analysis because it never completes its folding process without co-expression of the other members of the T-cell receptor complex. Instead of folding and trafficking along the secretory pathway, newly expressed CD3δ associates with CNX and accumulates in the ER for eventual degradation by ERAD (Klausner et al., 1990; Lippincott-Schwartz et al., 1988; van Leeuwen and Kearse, 1996). We plotted the fluorescence intensity of three YFP-PrP* NRK cells that were imaged every 15 m upon the induction of new CD3δ-CFP expression. Time traces of the fluorescence intensity revealed that after a ∼100 m lag period in which CD3δ-CFP accumulated (discussed below), incremental increases in the levels of CD3δ-CFP corresponded with incremental decreases in the levels of YFP-PrP* within the ER until a lower threshold of ER-localized YFP-PrP* was reached (Fig. 9, A and B; and Video 4).

**Video 4.** New expression of CNX-binding ERAD substrate, CD3δ-CFP, induces the release of YFP-PrP* from the ER for export to the Golgi. Time-lapse imaging of three representative YFP-PrP* FusRed-SiT NRK cells (Cells 1–3 from Fig. 9 A) transiently transfected with CD3δ-CFP. Images were collected every 15 min starting 2 h after transfection reagent with CD3δ-CFP expression plasmid was added. Scale bar, 10 μm.

To more directly test the hypothesis that the release of RESET substrates from CNX is regulated by competition with nascent PQC substrates, we took advantage of the tractable puromycinylation reaction (Schmidt et al., 2009). By adjusting the concentration of puromycin in the cell culture medium to generate more or less puromycinylated truncated protein products, we could test the impact of puromycinylated substrates on the interaction between RESET substrates such as YFP-PrP* and CNX within the ER. If the release of YFP-PrP* from CNX is regulated by competition, we predicted that the levels of CNX-associated puromycinylated substrates and YFP-PrP* would be inversely correlated. To minimize secondary effects with puromycin that may occur over time due to an eventual buildup of cytosolic truncation products, we incubated cells with puromycin for only 30 min, which was just long enough to detect an obvious association with CNX and relocalization of the YFP-PrP* from ER to Golgi (Fig. 8).

We purified endogenous CNX under non-denaturing conditions to preserve its interactions with associated proteins from YFP-PrP* NRK cells treated with 0, 1.25, or 2.50 µg/ml of puromycin for 30 min, performed western blots, and probed for CNX, YFP-PrP*, or puromycinylated proteins (Fig. 9 C). Experiments were performed as biological triplicates. Next, we quantified the relative amounts of the YFP-PrP* or puromycinylated proteins that coeluted with CNX across conditions and plotted the means and SD (Fig. 9 D). We addressed the variations in CNX pull-downs by normalizing the background-subtracted integrated densities of the YFP-PrP* bands or puromycinylated protein lanes against the background-subtracted integrated densities of the CNX bands. To facilitate the evaluation of the proportional changes between the samples, we performed a second round of normalization with respect to the highest/lowest levels of proteins (YFP-PrP* or puromycinylated proteins) that were pulled down. Thus, with the CNX-bound YFP-PrP* in untreated cells (i.e., cells treated with 0 µg/ml puromycin) normalized to 1, the relative values of CNX-associated YFP-PrP* in 0, 1.25, and 2.50 µg/ml puromycin-treated cells were 1, 0.88 ± 0.04 (SD), and 0.59 ± 0.09 (SD), respectively. The values for CNX-bound puromycinylated proteins were normalized so that the entire range was mapped between 0 (for the untreated sample) and 1 (for the 2.5 μg/ml puromycin-treated sample). The relative values of total CNX-associated puromycinylated proteins in 0, 1.25, and 2.50 µg/ml puromycin-treated cells were 0, 0.60 ±0.07, and 1 from 0, 1.25, and 2.50 µg/ml, respectively.

The inverse correlation between the levels of YFP-PrP* versus other PQC substrates that copurify with CNX (Fig. 5, C–E; and Fig. 9, C and D) implicate competition for CNX-binding as a major mechanism driving the release of misfolded GPI-APs from the ER to the Golgi.

## Discussion

**Figure S5.**
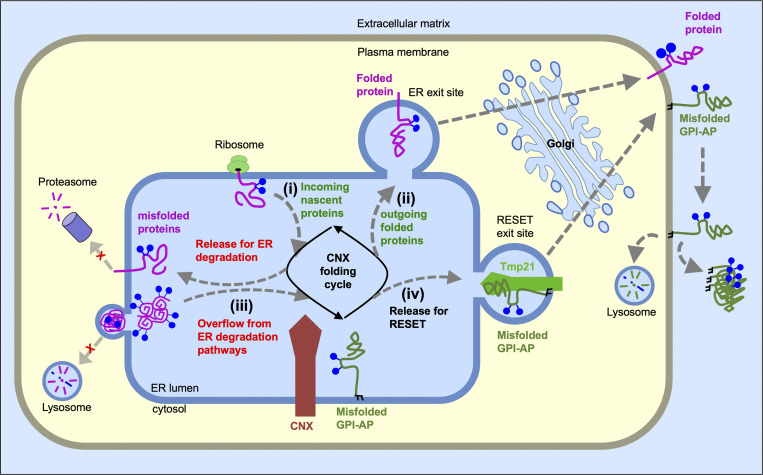
**Model depicting the role of CNX in regulating proteostasis of misfolded GPI-APs.** CNX functions as a traffic officer in the busy intersection of the ER, juggling incoming nascent proteins for folding and refolding cycles (i) and directing correctly folded proteins for ER-export and secretion (ii) or terminally misfolded proteins for ER clearance pathways (iii and iv). During steady-state conditions, newly synthesized proteins (i) displace misfolded GPI-APs from CNX and free them to be cleared by the RESET pathway (iv), which is mediated by Tmp21 and p24 family proteins. Once released to the cell surface via RESET, misfolded GPI-APs may be internalized for lysosomal degradation or potentially be deposited in the extracellular matrix. Thus, blocking new protein synthesis allows CNX to engage with misfolded GPI-APs indefinitely and extends their half-life. Conversely, upregulation of PQC substrates either through induction of new protein synthesis or through the inhibition or saturation of ER-localized degradation pathways (shown as red “x”s) overwhelms CNX, forcing CNX to rapidly release the misfolded GPI-APs for RESET and eventual secretion to the cell surface.

Misfolded GPI-APs are cleared from the ER by the RESET pathway for subsequent degradation in lysosomes (Satpute-Krishnan et al., 2014). Here, we set out to explain why ER-export of misfolded GPI-APs through the RESET pathway occurs constitutively and flux through the RESET pathway is increased during ER-stress, a condition wherein the load of PQC substrates exceeds the ER protein folding capacity (Hetz, 2012; Ron and Walter, 2007; Schröder and Kaufman, 2005). We took a multipronged approach to acutely perturb the balance between PQC substrates and key PQC machinery (i.e., CNX) using global inhibitors (i.e., NMS-873, MG-132, DNJ, puromycin, cycloheximide, or anisomycin) or overexpression of diverse individual glycoproteins that bind to CNX (i.e., CD3d, Thy-1, IL-6, vIL-6, or SARS Cov2-Spike). The results from each approach fit with a competition model in which the association of new substrates with CNX promotes the release of YFP-PrP*’s from CNX for constitutive turnover via RESET (Fig. S5). The competition model provides a concise explanation for why inhibiting or escalating the production of CNX substrates, respectively, slows down or speeds up the flux of misfolded GPI-APs through the RESET pathway.

A key finding from this study is that RESET substrates remain engaged with CNX in the ER until a new folding substrate engages with CNX. This discovery contributes to the overall understanding of CNX’s multifaceted involvement in GPI-AP biogenesis and PQC. The van der Goot group uncovered a role for CNX in the cotranslational translocation of PrP (Lakkaraju et al., 2012). Studies performed by Kinoshita, Fujita, and colleagues established that CNX interacts with newly synthesized GPI-APs via CNX’s lectin domain and that interaction with CNX is crucial for allowing for GPI-anchor inositol deacylation, a prerequisite step for efficient ER export of properly folded GPI-APs for secretion or misfolded GPI-APs for RESET (Guo et al., 2020; Liu et al., 2018). Our demonstration that competition between nascent CNX substrates and misfolded GPI-APs drives constitutive release of misfolded GPI-APs for turnover highlights dissociation from CNX as a critical step in the PQC of misfolded GPI-APs.

The cellular proteome is in a constant state of fluctuation and adaptation in response to changing internal and external environments (Harper and Bennett, 2016; Jayaraj et al., 2020). The findings presented here offer new insights into how competitive interactions between nascent proteins and RESET substrates impact the dynamic equilibrium of the proteomic milieu across the secretory pathway. Important follow-ups to this study will be to delineate at a molecular level which of many possible mechanisms drive the competition between nascent proteins and existing RESET substrates for CNX binding. Possible mechanisms include the ability of nascent proteins to directly displace RESET substrates or indirectly displace RESET substrates from CNX by inducing a change in CNX’s physical (e.g., structural or heterooligomeric) state or by associating with CNX’s partners in protein folding and PQC. Proteomics approaches may reveal additional ER PQC factors that work in conjunction with CNX to retain RESET substrates in the ER. Finally, the lag time between CD3δ buildup in the ER and YFP-PrP* release that was observed in Fig. 9 A brings up the question of whether CNX (alone or in conjunction with other ER PQC chaperones) has the capacity to buffer or absorb moderate increases in PQC substrates within a certain range. The work presented here provides the first demonstration that the new induction of glycoproteins, as occurs during inflammation or envelope virus infection, promotes the release of misfolded GPI-APs from CNX for RESET.

We demonstrate here that acute CNX depletion instantly frees misfolded GPI-APs to undergo RESET. This is consistent with demonstrations by others that interfering with binding between CNX’s lectin domain and GPI-APs’ monoglucosylated glycans is sufficient to release GPI-APs for ER-export (Guo et al., 2020; Liu et al., 2018). Additionally, this is consistent with our previous finding that thapsigargin or dithiothreitol-induced dissociation of YFP-PrP* from CNX correlated with the release of at least 85% of the YFP-PrP* from the ER to the Golgi within 30 min, followed by near complete degradation of YFP-PrP* within 60 min in NRK cells (Satpute-Krishnan et al., 2014). Using time-lapse imaging of YFP-PrP*, we found that diverse cell culture systems including NRK, N2a, HeLa, Cos-7, IB3-1, and HEK293 demonstrate similar kinetics for rapid thapsigargin-induced ER-export of YFP-PrP* to the Golgi (Satpute-Krishnan et al., 2014). In comparison, the kinetics of thapsigargin-induced ER-export of GFP-PrP* in the HEK293 Flp-InT-REx cell line were relatively delayed with little to no degradation within 60 min of the addition of thapsigargin (Zavodszky and Hegde, 2019). The discrepancy in kinetics between ER export of PrP* in HEK293 Flp-InT-REx cells as compared with the panel of cells tested previously may be a consequence of the fundamental difference in the cell culture model system. Intriguingly, Zavodszky and Hegde demonstrated that a contingent of CNX, comprising ∼0.5% of total cellular CNX, trafficked together with GFP-PrP* from the ER to the cell surface prior to internalization in lysosomes in the thapsigargin-treated HEK293 Flp-InT-REx cells (Zavodszky and Hegde, 2019). This, along with reports of a small fraction of CNX appearing on the cell surface of other cell types (Okazaki et al., 2000; Wiest et al., 1995), evokes future studies to determine how CNX export is regulated across cell types and in thapsigargin-treated conditions but does not conflict with our proposed model that the major mechanism for release of RESET substrates during steady-state conditions is competition for binding to a larger, ER-localized pool of CNX.

Unlike conditions that induce RESET through the buildup of CNX substrates, this study suggests that thapsigargin or dithiothreitol triggers RESET of misfolded GPI-APs through a different mechanism. MG132, NMS-873, DNJ, and puromycin-induced RESET each correlated with the buildup of ER PQC substrates and were each blocked with the addition of protein translation inhibitors, while thapsigargin and dithiothreitol-induced RESET were not affected by protein translation inhibitors (Fig. 1 H and Fig. S3 C). Additionally, treatment with DNJ caused an increase in N-linked glycosylated proteins that coeluted with CNX, while thapsigargin and dithiothreitol did not (Fig. 5 C). The near-instantaneous effect of thapsigargin or dithiothreitol to induce RESET may be due to a structural alteration in CNX. Other groups have shown that dithiothreitol treatment of cells correlated with the reduction of disulfide bonds within CNX and the release of CNX substrates (Hebert et al., 1995; Tector and Salter, 1995; Vassilakos et al., 1998). Likewise, calcium-binding was proposed to impact CNX’s association with substrates (Di Jeso et al., 2003; Wada et al., 1991), and thapsigargin-induced depletion of ER calcium was shown to impact CNX’s structure (Ihara et al., 1999; Ou et al., 1995; Thammavongsa et al., 2005). Similarly, in the case of an unrelated ER chaperone, BiP, thapsigargin was shown to induce a structural change that correlated with the rapid release of TCR alpha for ER-export (Preissler et al., 2020; Suzuki et al., 1991). While there is precedence for the idea that acute ER-stressors like thapsigargin or dithiothreitol accelerate the degradation of PQC substrates (Jung et al., 2015; Wileman et al., 1991), future studies to determine precisely how these commonly used chemical ER stressors impact individual PQC factors will expand our insight into what these chemicals reveal about the proteostasis network. This is exemplified by the discovery presented here that cycloheximide, a chemical that is routinely used to measure protein half-life, itself delays the degradation of RESET substrates.

## Materials and methods

### Generation of stable cell lines and cell culture

The isolation of the clonal line of Normal Rat Kidney (NRK) cells used here was described previously (Hailey et al., 2010). Creation of NRK cell lines stably expressing YFP-PrP C179A (YFP-PrP* NRK cells), YFP-CD3δ, and GFP-CD59 C94S were described previously (Satpute-Krishnan et al., 2014). Critically, YFP-PrP* NRK cells were sorted for an expression level of YFP-PrP* similar to wild-type PrP from mouse brain homogenate, as previously described (Satpute-Krishnan et al., 2014), and demonstrated here (Fig. S1). YFP-CD3δ and GFP-CD59 C94S were expressed at similar levels to YFP-PrP* in the stably transfected cell lines (data not shown). N2a refers to mouse neuro-2a cells (ATCC CCL-121).

To generate TetOne Nixin-Cerulean YFP-PrP* stable NRK cells, YFP-PrP* NRK cells were transfected with the TetOne Nixin-Cerulean plasmid (described below) and selected with 2 μg/ml puromycin (Sigma-Aldrich) for 1 wk. Nixin-Cerulean was induced overnight with 0.5 μg/ml of doxycycline and on the next morning cells were subjected to one round of sorting for CFP and YFP fluorescence resulting in a population of cells enriched to ∼50% cells for TetOne Nixin-Cerulean YFP-PrP* expressing NRK cells. For western blot experiments, Nixin-Cerulean was induced with 0.5 μg/ml of doxycycline for 8 h prior to harvesting cells.

To generate YFP-PrP* Cer-GalT NRK cells, YFP-PrP* NRK cells were transfected with the Cer-GalT plasmid (gift from Dr. Jennifer Lippincott-Schwartz, HHMI Janelia Research Campus, Ashburn, VA, USA; plasmid #11930; Addgene [Cole et al., 1996]) and selected with 300 mg/ml G418 Sulfate (AG Scientific) for 1 wk prior to sorting for CFP and YFP double transfectants.

NRK cell lines or N2a cells were maintained in complete culture medium DMEM (Corning) supplemented with 10% fetal bovine serum (Corning) and 2 mM *L*-glutamine (Corning) at 37°C with 5% CO_2_.

Transfections of NRK cell lines or N2a cells were done at 60–70% confluency using Lipofectamine 3000 (Invitrogen). For all experiments involving transient transfections, unless specified otherwise, experiments were performed 48–72 h after transfection. To allow enough time for cells that were massively overexpressing the plasmid-encoded protein to die and be washed away, we consistently split transiently transfected cells 1:2 around 24 h after transfection onto coverslips or dishes for imaging or column purification experiments, respectively. The experiments were performed between 48 and 72 h after transfection.

### Generation of plasmids and plasmid attributions

Construction details of YFP-PrP*, YFP-PrP M129V F198S, and GFP-CD59 C94S were previously described (Satpute-Krishnan et al., 2014). The following expression constructs were described previously: Cer-GalT plasmid (gift from Dr. Jennifer Lippincott-Schwartz, HHMI Janelia Research Campus, Ashburn, VA, USA; plasmid #11930; Addgene [Cole et al., 1996]); CD3δ-CFP plasmid (gift from Dr. Jennifer Lippincott-Schwartz, HHMI Janelia Research Campus, Ashburn, VA, USA; plasmid #128154; Addgene [Lorenz et al., 2006]); ER-mCFP-KDEL (gift from Dr. Erik Snapp, HHMI Janelia Research Campus, Ashburn, VA, USA, constructed in parallel to ER-mGFP-KDEL [Snapp et al., 2006]); LAMP1-mCherry (LAMP1 with C-terminal fluorescent protein tag; gift from Dr. George Patterson, National Institutes of Health, Bethesda, MD, USA, constructed exactly as described for PA-GFP-lgp120 [Patterson and Lippincott-Schwartz, 2002]); and FusionRed-SiT-15 (gift from Dr. Michael W. Davidson, Florida State University, Tallahassee, FL, USA; plasmid #56133; Addgene [Shemiakina et al., 2012]).

Cer-Thy1 was generated by replacing the GFP tag in ss(lactase)-GFP-Thy1 construct, previously described (Satpute-Krishnan et al., 2014), with Cerulean in the AgeI and BsrGI restriction sites. “ss(lactase)” refers to rabbit lactase-phlorizin hydrolase signal sequence. Thy1 refers to the “mature domain” of Thy1 (i.e., the open reading frame sans the signal sequence). Both Cer-IL-6 and Cer-vIL-6 were constructed by replacing the Thy1 with IL-6/vIL-6 mature domains using NEBuilder. For Cer-IL-6, IL-6 was PCR amplified from IL-6 (human) ORF (Genscript) and the ss(lactase)-Cer and vector from ss(lactase)-Cer-Thy1 prior to assembly. To PCR amplify the ss(lactase)-Cer and vector sequence, leaving behind Thy1, these primers were used: (forward) 5′-CTC​GAG​GGA​TCT​TCC​ATA​C-3′ and (reverse) 5′-GCTGCCTCCTGC CTTGTA-3′. To PCR amplify IL-6 with overhangs that annealed with the ss(lactase)-Cer vector, these primers were used: (forward, IL-6) 5′-CGA​GCT​GTA​CAA​GGC​AGG​AGG​CAG​CGT​ACC​CCC​AGG​A GAAGAT-3′ and (reverse, IL-6) 5′-CTG​GTA​GGT​ATG​GAA​GAT​CCC​TCG​AGT​TAT​CAC​TTA​TCG​TCG​TCA​TC-3′. To create ss(lactase)-Cer-vIL6(human), the same ss(lactase)-Cer vector backbone was assembled with the following custom GeneStrand (Eurofins) containing vIL-6 flanked by vector-annealing sequence: 5′-CGA​GCT​GTA​CAA​GGC​AGG​AGG​CAG​CAA​GCT​GCC​CGA​CGC​CCC​CGA​GTT​CGA​GAA​GGA​CCT​GCT​GAT​CCA​GAG​ACT​GAA​CTG​GAT​GCT​GTG​GGT​GAT​CGA​CGA​GTG​CTT​CAG​AGA​CCT​GTG​CTA​CAG​AAC​CGG​CAT​CTG​CAA​GGG​CAT​CCT​GGA​GCC​CGC​CGC​CAT​CTT​CCA​CCT​GAA​GCT​GCC​CGC​CAT​CAA​CGA​CAC​CGA​CCA​CTG​CGG​CCT​GAT​CGG​CTT​CAA​CGA​GAC​CAG​CTG​CCT​GAA​GAA​GCT​GGC​CGA​CGG​CTT​CTT​CGA​GTT​CGA​GGT​GCT​GTT​CAA​GTT​CCT​GAC​CAC​CGA​GTT​CGG​CAA​GAG​CGT​GAT​CAA​CGT​GGA​CGT​GAT​GGA​GCT​GCT​GAC​CAA​GAC​CCT​GGG​CTG​GGA​CAT​CCA​GGA​GGA​GCT​GAA​CAA​GCT​GAC​CAA​GAC​CCA​CTA​CAG​CCC​CCC​CAA​GTT​CGA​CAG​AGG​CCT​GCT​GGG​CAG​ACT​GCA​GGG​CCT​GAA​GTA​CTG​GGT​GAG​ACA​CTT​CGC​CAG​CTT​CTA​CGT​GCT​GAG​CGC​CAT​GGA​GAA​GTT​CGC​CGG​CCA​GGC​CGT​GAG​AGT​GCT​GAA​CAG​CAT​CCC​CGA​CGT​GAC​CCC​CGA​CGT​GCA​CGA​CAA​GTG​ATA​ACT​CGA​GGG​ATC​TTC​CAT​ACC​TAC​CAG-3′. CER-SARS-CoV-2 Spike Glycoprotein “CER-SARS2-Spike” was generated from pcDNA3.1-SARS2-Spike (145032; Addgene; Shang et al., 2020) and assembled with NEBuilder. To PCR-amplify SARS2-Spike with overhangs that annealed with the ss(lactase)-Cerulean vector from CER-Thy1, these primers were used: (forward 1, SARS2-Spike) 5′-CGA​GCT​GTA​CAA​GGC​AGG​AGG​CAG​CCA​GTG​CGT​GAA​CCT​GAC​TA-3′, (forward 2, SARS2-Spike) 5′-CCA​ACG​TGT​TCC​AGA​CCC-3′ and (reverse 1, SARS2-Spike) 5′-GTT​CAC​GTG​CTC​AGC​TCC-3′, (reverse 2, SARS2-Spike) 5′-CTG​GTA​GGT​ATG​GAA​GAT​CCC​TCG​AGT​CAT​GTA​TAG​TGC​AGT​TTG​ACG​C-3′.

Nixin-CER was created by replacing the YFP with Cerulean into the AgeI and BsrGI cut sites of Nixin-YFP (gift from Dr. Albert Neutzner, University of Basel, Basel, Switzerland [Neutzner et al., 2011]). To create doxycycline-inducible Nixin-CER (“TetOne Nixin-CER”), Nixin-CER open reading frame was PCR-amplified with primers including pLVX-TetOne-Puro vector-annealing overhangs: (forward) 5′-CAC​TTC​CTA​CCC​TCG​TAA​AGA​TGC​CGC​TCT​GCC​GTC​CGG-3′ and (reverse) 5′-CTC​GCA​GGG​GAG​GTG​GTC​TGT​TAC​TTG​TAC​AGC​TCG​TCC​A-3′. pLVX-TetOne-Puro vector (Takara) was linearized by cutting at EcoRI and BamH1 restriction sites and assembled with Nixin-CER using NEBuilder.

GFP-CNX and SNAPflag-CNX were both made with CNX human open reading frame (HsCD00042644; DNASU). To make GFP-CNX, the Thy1 mature domain in ss(lactase)-GFP-Thy1 was replaced with CNX mature domain using NEBuilder, similar to the construction of CER-IL-6, using primers with vector overhangs: (forward) 5′-GAG​CTG​TAC​AAG​GCA​GGA​GGC​AGC​CAT​GAT​GGA​CAT​GAT​GAT​GAT-3′ and (reverse) 5′-TGG​TAG​GTA​TGG​AAG​ATC​CCT​CGA​GCT​ACT​CTC​TTC​GTG​GCT​TTC-3′. To make SNAPflag-CNX, the vector sequence of CER-CNX sans the Cerulean tag was amplified with primers (forward) 5′-ATG​GAC​AAA​GAC​TGC​GAA​AT-3′ and (reverse) 5′-CTT​GTC​ATC​GTC​GTC​CTT​G-3′, and replaced the CER with SNAPflag that was PCR amplified from pME-puro-SNAP-FLAG-CD59 (gift from Dr. Reika Watanabe, La Jolla Institute for Immunology, San Diego, CA; Addgene plasmid #50374) using the following primers with overhangs for the vector: (forward) 5′-GAC​TAC​AAG​GAC​GAC​GAT​GAC​AAG​GCA GGAGGCAGCCATGA-3′, and (reverse) 5′-GCT​TCA​TTT​CGC​AGT​CTT​TGT​CCA​TGG​TGG​CGA​CCG​GTG​GA-3′.

### Antibodies and lectin blotting

For western blot analysis, the following commercial primary antibodies were used to probe for calnexin (SPA-860, rabbit polyclonal; StressGen), calreticulin (SPA-602, rabbit polyclonal; StressGen), Erp29 (67675-1-IG, mouse monoclonal; Proteintech), ERp57 (23886, mouse monoclonal; Santa Cruz), GAPDH (MA5 15738, mouse monoclonal; Thermo Fisher Scientific), GFP (66002-1-IG, mouse monoclonal; Proteintech), Glucosidase1/MOGS (17859-1-AP, rabbit polyclonal; Proteintech), Glucosidase 2/PRKCSH (12148-1-AP, rabbit polyclonal; Proteintech), GRP78/BiP (AF4846, goat polyclonal; R&D), GRP94 (SPA-850, rat monoclonal; StressGen), Malectin (26655-1-AP, rabbit polyclonal; Proteintech), Nixin (H00148066-B01P, mouse polyclonal; Novus Bio), and UGGT1/UGCGL1 (14170-1-AP, rabbit polyclonal; Proteintech). Homemade rabbit antisera for Tmp21 was previously described (Satpute-Krishnan et al., 2014). The following secondary antibodies were used: HRP-conjugated goat IgG antibody (HAF109; R&D), HRP-conjugated goat anti-mouse IgG (H+L) Superclonal Secondary antibody (A28177; Thermo Fisher Scientific), HRP-conjugated goat anti-rabbit IgG (H+L) Superclonal Secondary antibody (A27036; Thermo Fisher Scientific).

For steady-state chases, homemade rabbit antisera for GFP and RFP, which were gifts from Dr. Jennifer Lippincott-Schwartz, HHMI Janelia Research Campus, Ashburn, VA, were used as previously described (Satpute-Krishnan et al., 2014).

For immunofluorescence of calnexin, anti-calnexin primary (AB22595; Abcam, rabbit polyclonal) and Cy5-conjugated goat anti-rabbit IgG (111-175-003; Jackson ImmunoResearch) were used.

For lectin blotting of N-linked glycosylated proteins, HRP-conjugated wheat germ agglutinin was used per the manufacturer’s instructions (L3892; Sigma-Aldrich).

For antibody uptake imaging experiments, we used Alexa Fluor 647-conjugated rabbit anti-GFP antibody (A-31852; Thermo Fisher Scientific).

### Drug treatments

The following reagents were used at working concentrations as listed: 50 μg/ml cycloheximide (C-1189; AG Scientific), 10 μM MG-132 (474791; Sigma-Aldrich), 10 μM NMS-873 (6180/25; R&D), 0.5 μM anisomycin (A9789; Sigma-Aldrich), 2.5 mM deoxynojirimycin (Cayman Chemicals), 0.1 μM thapsigargin (586005; EMD), 0.5 mM 1,3-dithiothreitol (10708984001; Roche), 250 nM bafilomycin A1 (B-1080; LC Laboratories), 0.5 μg/ml doxycycline (D9891; Sigma-Aldrich), 2 μg/ml puromycyin (P9620; Sigma-Aldrich), and 300 mg/ml G418 sulfate (G-1033; AG Scientific).

### Steady-state chase assay

Steady-state chase experiments were performed in stably transfected NRK cells cultured in six-well dishes to ∼60–80% confluency and labeled for 12–13 h with 100 μCi/ml 35S label (NEG772007MC; Perkin Elmer) in Cys-free/Met-free media (D0422; Sigma-Aldrich) supplemented with 10% fetal bovine serum (FBS), 2 mM *L*-Glu, 0.6 μM Cys, and 2 μM Met. For the chase, cells were washed with PBS to remove the label and incubated with the complete culture medium (described above). Drugs were added at the time of chase. Finally, cells were washed in PBS and solubilized in 150 μl of 1% SDS, 0.1 M Tris, pH 8 by boiling/vortexing. Lysates were diluted 10-fold in immunoprecipitation (IP) buffer (1% Triton X-100, 50 mM HEPES, pH 7.4, 100 mM NaCl) on ice and clarified at 4°C by centrifugation in a microcentrifuge. Immunoprecipitation was done by incubating lysates with specified rabbit polyclonal antibodies and protein A gel (153-6154; Bio-Rad). Proteins were separated on 10 or 12% Mini-PROTEAN TGX precast protein gels. The gels were dried and exposed to a storage phosphor screen (2025 28-9564-75; GE BAS-MS), which was subsequently scanned on an Amersham Typhoon RBG. To verify equal starting material and equal recovery of the antibody complexes, diluted lysates were also analyzed and gels of the IP were stained with Coomassie Brilliant Blue R-250 dye (Bio-Rad) to visualize antibody light and heavy chains.

### Column purifications

GFP and DYKDDDDK (Flag) pull-downs were performed 48 h after transient transfection of N2a cells that were cultured in 10-cm dishes to 60–80% confluency by the μMACS GFP Isolation Kit (130-091-288; Miltenyi Biotec) and μMACS DYKDDDDK Isolation Kit (130-101-591; Miltenyi Biotec). Pull-downs of endogenous CNX were performed in YFP-PrP* NRK cells that were cultured in 10-cm dishes to ∼70% confluency using μMACS Protein A Kit (130-092-945; Miltenyi Biotec) combined with rabbit anti-CNX antibody (SPA-860; Enzo). All pull-downs were performed exactly according to the manufacturer’s recommendations, with one exception: a homemade lysis buffer, which was optimized to maintain protein–protein interactions with calnexin (Satpute-Krishnan et al., 2014), comprising 1% CHAPS, 50 mM HEPES, pH 7.4, 100 mM NaCl, and 2 mM CaCl_2_ and was used for cell lysis, magnetic antibody-conjugated microbead incubation, and washes on the magnetic column. The entire column purification procedure prior to elution was performed in a 4°C cold room and on ice to stabilize interactions. Apyrase was not included in any of the column purification protocols.

### SDS-PAGE and western blot

Samples were collected in 2X Laemmli sample buffer (Bio-Rad) and boiled and vortexed. Proteins were separated on 12% or 4–20% TGX Pre-Cast Stain-free gels (Bio-Rad). The Bio-Rad Stain-free gel system was used to show input for the loading control. Gels were transferred onto PVDF membranes using the Trans-Blot Turbo Transfer System and blocked with 5% non-fat dry milk in TBS + 0.1% Tween (TBST). Primary and HRP-conjugated secondary antibodies were incubated on a shaker in 5% non-fat dry milk in TBST and washed three times in TBST after each incubation. Membranes were submerged in Clarity or Clarity Max enhanced chemiluminescence substrate (Bio-Rad) for 1–5 min, and chemiluminescence was imaged with a Bio-Rad ChemiDoc system.

### Quantification and statistical analyses of western blots

For western blot and pulse-chase bands, relative pixel density values from three independently performed experiments were presented as mean ± SD (*n* = 3) in bar plots, as indicated in the figure legends. Means and SD were calculated and plotted using Microsoft Excel.

For densitometry analysis of western blot or pulse-chase bands, ImageJ was used to measure relative pixel densities of bands on unsaturated images collected as eight-bit .tif files from the Chemi-Doc (Bio-Rad). A rectangular ROI sized to enclose the band with the largest area was created and the same ROI was used to measure the integrated density of the rest of the bands. Integrated density values were background subtracted and normalized against “time 0” or “untreated” bands to obtain relative pixel densities. Quantification details for specific experiments are provided in the adjoining figure legends.

### Confocal microscope imaging experiments

Images and time-lapse series were collected with Nikon inverted spinning disk confocal microscope equipped with Yokogawa CSU-X1 Spinning Disk, EMCCD camera, laser launch including 445 nm (to image Cerulean and CFP), 488 nm (to image GFP), 514 nm (to image Venus and YFP), 561 nm (to image mCherry), and 647 nm (to image Cy5), focus drift correction through the Perfect Focus System, and 60× Plan Apo 1.40 NA oil/0.13 mm WD, and controlled by the NIS-Elements software. For live-cell imaging experiments, cells were seeded in # 1.5 coverslip bottom dishes and incubated in a complete DMEM cell culture medium (described above) at 37°C with 5% CO_2_ within a Tokai incubator on the microscope stage. Imaging experiments were performed at 60–80% confluency of stably transfected cells or 48–72 h after transient transfection, unless otherwise specified in the figure legend.

### Live cell anti-GFP antibody uptake imaging experiments

YFP-PrP* NRK or parental NRK cells were transfected with Lamp1-mCherry using Lipofectamine 3000. 24 h after transfection, cells were seeded onto #1.5 glass coverslips. 48 h after transfection, cells were incubated with 0.4 μg/ml Alexa Fluor 647-conjugated anti-GFP antibody (A-31852; Thermo Fisher Scientific) and 250 nM Bafilomycin A1 (B-1080; LC Laboratories) for time-lapse imaging. Where indicated, 2.5 mM deoxynojirimycin (Cayman Chemicals) was included in the medium.

### siRNA knockdown of Tmp21

Tmp21 was depleted by two sequential transfections 48 h apart of the ON-TARGETplus SMARTpool Rat Tmed10 siRNA (L-092937-01-0005; Dharmacon) with Dharmafect 2 transfection reagent (T-2002-02; Dharmacon) exactly as recommended by the manufacturer. Immediately after the second siRNA transfection, cells were transferred to coverslips, and 24 h later, the imaging experiments were performed.

### Acquisition, processing, and analysis of confocal images

Microscopy images were originally acquired into NIS-Elements (Nikon) and processed to split or merge channels before exporting image stacks in .tif format for figures or full-resolution (no compression) movies in .avi format. FIJI (ImageJ; National Institutes of Health [NIH]) was used to adjust brightness and contrast, overlay the scale bar and labels, and indicate the elapsed time with the Time Stamper tool. FIJI was used to export the annotated image stacks to eight frame-per-second videos with JPEG compression. For figure preparation, Adobe Photoshop was used to arrange images into panels and linearly adjust intensity levels to optimize brightness and contrast. No non-linear adjustments were made.

Green and red channel intensity plot profiles were created using ImageJ (NIH) with the RGB Profiler ImageJ plugin (https://imagej.net/RGB_Profiler) provided for public use by Christophe Laummonerie and Jerome Mutterer.

The changes in concentration of YFP-PrP* and CD3δ-CFP in the ER following transient transfection of cells with CD3δ-CFP were quantified by evaluating the fluorescence intensities of the two proteins in the ER (i.e., within pixels belonging to ER) at each time-point of the image time series. To do this, initially, binary masks of ER and Golgi for each time point of the image series were generated. The ER at each time-point of the image series was identified either by the YFP-PrP* fluorescence signal (in the initial frames immediately after transfection of CD3δ) or by the CD3δ-CFP fluorescence signal (once the CD3δ-CFP fluorescence has increased sufficiently for segmentation analysis). The YFP-PrP*/CD3δ-CFP images were subjected to an image segmentation algorithm to generate initial binary masks of the ER $(Mask_{ER}^{Initial})$. Golgi masks $({Mask}_{Golgi})$ corresponding to each of the ER masks were subsequently generated by image segmentation of the SiT-FusRed images. Next, to remove the contribution of fluorescence signal from Golgi to the measured signal (since some portions of Golgi spatially overlap with ER in the images), a second set of ER-masks were generated, which only marked pixels that exclusively belong to ER. This was done by excluding from each ER mask the pixels that overlapped with the corresponding Golgi mask $\left(Mask_{ER}^{Final}=Mask_{ER}^{Initial}-{Mask}_{Golgi}\right)$. In the resulting ER-mask images, pixels corresponding exclusively to the ER (but not overlapping with Golgi) have a value of 1, whereas all other pixels have a value of 0. Finally, the total background-corrected fluorescence of YFP-PrP* and CD3δ-CFP within the pixels belonging to the final ER-masks was calculated for each time point of the image series. The normalized values YFP-PrP* and CD3δ-CFP fluorescence in the ER were then plotted against time to visualize their concentration changes in the ER following transfection of CD3δ-CFP. The image analysis was performed using MATLAB (MathWorks) and ImageJ (NIH).

### Online supplemental material

Fig. S1 shows a western blot for prion protein (PrP) comparing PrP levels in lysates from a mouse brain and cultured YFP-PrP* NRK cells. Fig. S2 shows the effect of cycloheximide on GFP-CD59 C94S turnover and trafficking via RESET, respectively, as visualized by (A) an autoradiograph of a steady-state chase and (B) time-lapse imaging series of cells cotreated with MG132. Fig. S3 shows the effect of deoxynojirimycin (DNJ), thapsigargin (TG), or dithiothreitol (DTT) alone or with cycloheximide (CHX) on trafficking of misfolded GPI-APs through time-lapse imaging of (A) N2a cells expressing YFP-PrP* and treated with DNJ or CHX+DNJ, (B) NRK cells stably expressing GFP-CD59 C94S and treated with DNJ or CHX+DNJ, or (C) YFP-PrP* CER-GalT NRK cells treated with TG or CHX+TG, DTT or CHX+DTT. Fig. S4 shows the effect of new CD3δ-CFP expression on the trafficking of YFP-PrP* through time-lapse imaging. Fig. S5 shows a model depicting the role of CNX in regulating proteostasis of misfolded GPI-APs. Video 1 shows an expanded time-lapse imaging sequence of the still frames from Fig. 3 (C) of YFP-PrP M129V F198S and CER-GalT co-expressing NRK cells that were incubated with NMS-873. Video 2 shows an expanded time-lapse imaging sequence of the antibody uptake assay shown in Fig. 6 (B) of YFP-PrP* and Lamp1-mCherry co-expressing NRK cells or Lamp1-mCherry expressing NRK cells that were treated with bafilomycin and AlexaFluor-conjugated antibodies. Video 3 shows an expanded time-lapse imaging sequence of the antibody uptake assay shown in Fig. 6 (C) of YFP-PrP* and Lamp1-mCherry co-expressing NRK cells that were treated with DNJ, bafilomycin, and AlexaFluor-conjugated antibodies. Video 4 shows time-lapse imaging of the three representative YFP-PrP* FusRed-SiT NRK cells (Cells 1–3 from the plots in Fig. 9 A) after transfection with CD3δ-CFP.

## Supplementary Material

SourceData F1 is the source file for Fig. 1.

SourceData F4 is the source file for Fig. 4.

SourceData F5 is the source file for Fig. 5.

SourceData F7 is the source file for Fig. 7.

SourceData F8 is the source file for Fig. 8.

SourceData F9 is the source file for Fig. 9.

SourceData FS1 is the source file for Fig. S1.

SourceData FS2 is the source file for Fig. S2.
