# Supplementary material for: Competition for calnexin binding regulates secretion and turnover of misfolded GPI-anchored proteins
Source: J Cell Biol. 2023 Sep 13;222(10):e202108160. doi: 10.1083/jcb.202108160 (PMC10499038; doi:10.1083/jcb.202108160)
Supplement: SourceData F1 — is the source file for Fig. 1. [file JCB_202108160_SourceDataF1.pdf]

FIGURE 1 B

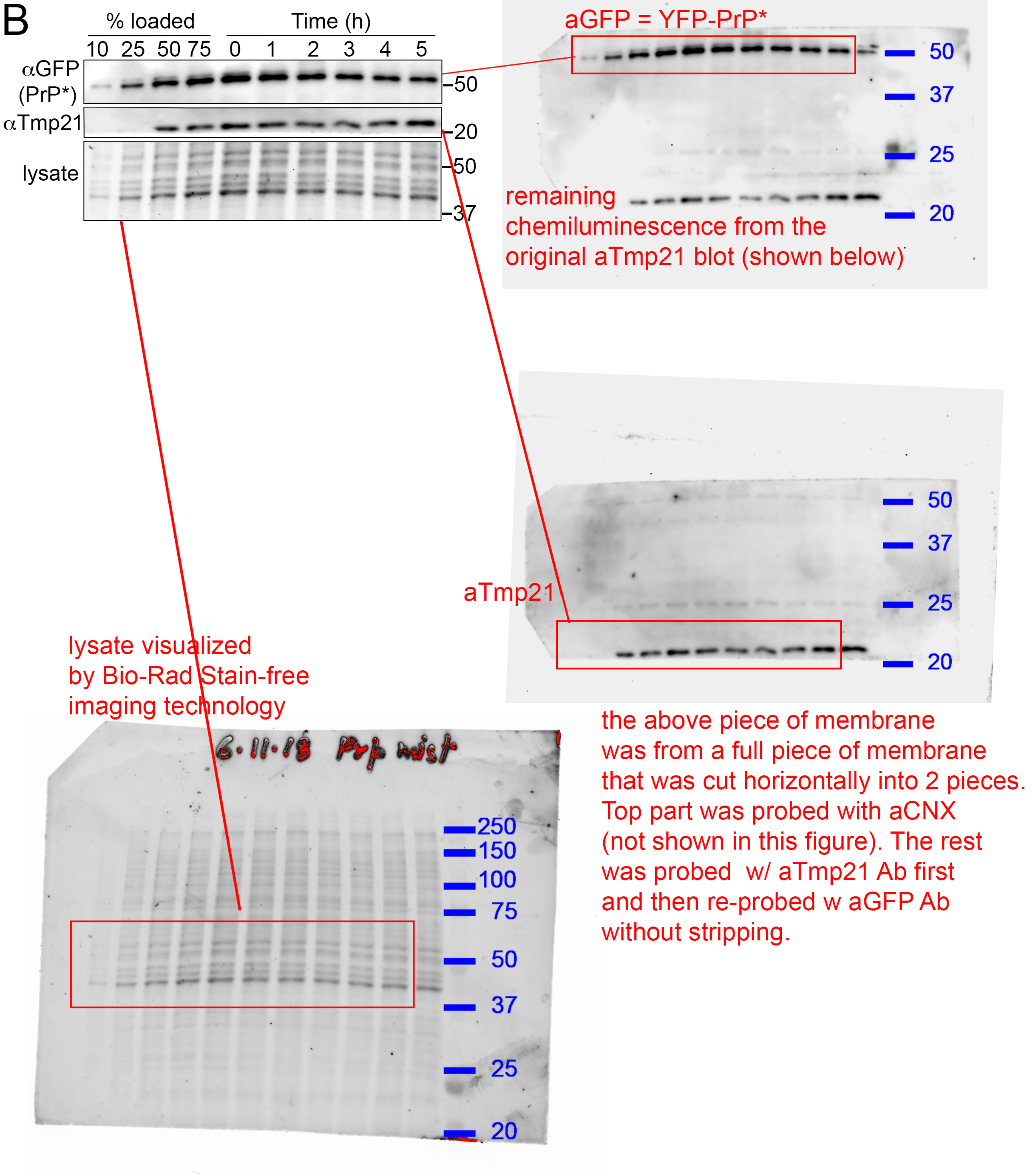
B
% loaded
Time (h)
10 25 50 75 0 1 2 3 4 5
αGFP (PrP*)
αTmp21
lysate
-50
-20
-50
-37
aGFP = YFP-PrP*
50
37
25
20
remaining chemiluminescence from the original aTmp21 blot (shown below)
aTmp21
50
37
25
20
lysate visualized by Bio-Rad Stain-free imaging technology
250
150
100
75
50
37
25
20
the above piece of membrane was from a full piece of membrane that was cut horizontally into 2 pieces. Top part was probed with aCNX (not shown in this figure). The rest was probed w/ aTmp21 Ab first and then re-probed w aGFP Ab without stripping.

FIGURE 1 C

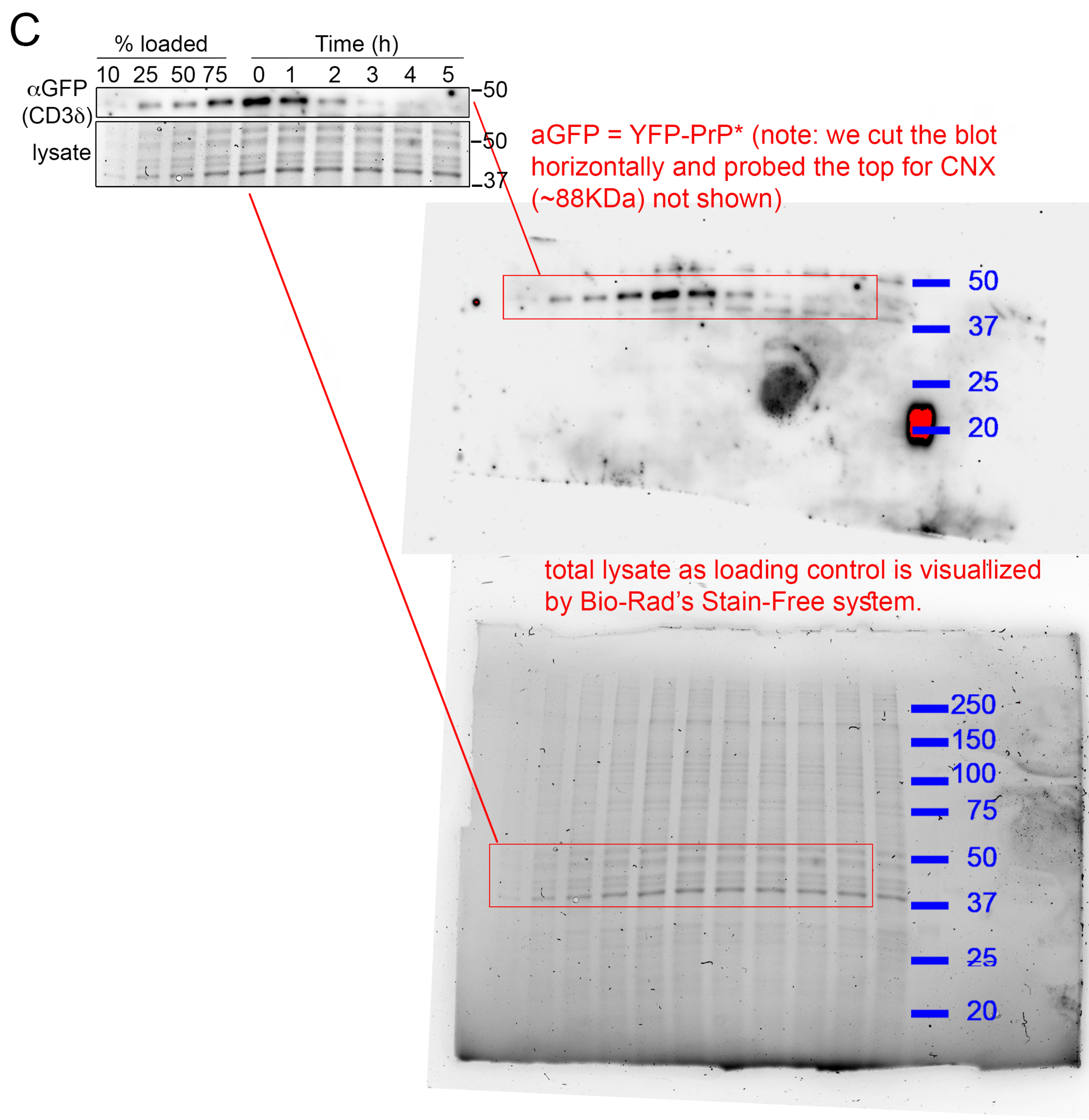

C
% loaded
Time (h)
10 25 50 75 0 1 2 3 4 5
αGFP
(CD3δ)
lysate
50
50
37
aGFP = YFP-PrP* (note: we cut the blot horizontally and probed the top for CNX (~88KDa) not shown)
50
37
25
20
total lysate as loading control is visualized by Bio-Rad's Stain-Free system.
250
150
100
75
50
37
25
20

FIGURE 1 E

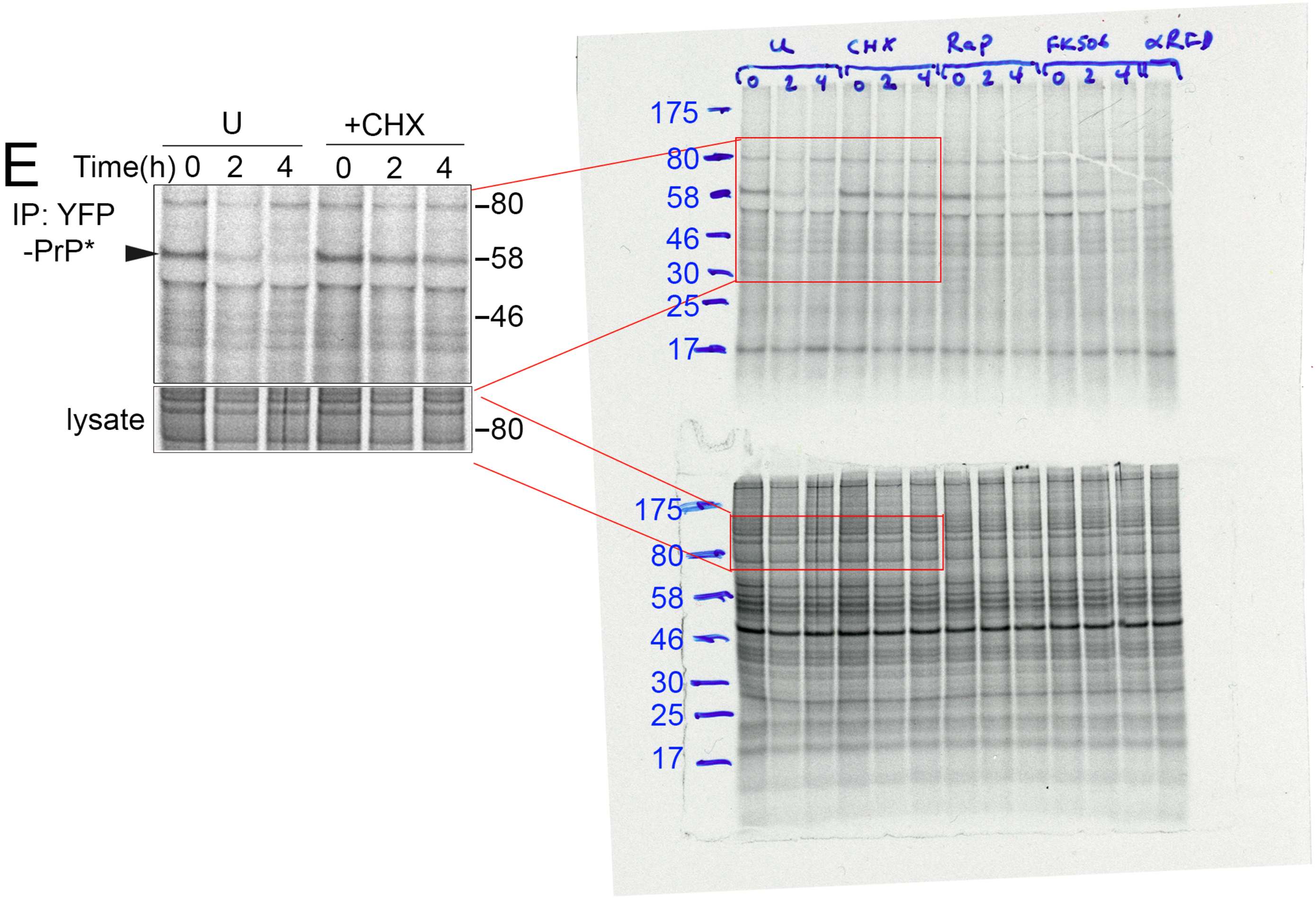
E
U
+CHX
Time(h) 0 2 4 0 2 4
IP: YFP
-PrP*
–80
–58
–46
lysate
–80
175
80
58
46
30
25
17
175
80
58
46
30
25
17
