# Supplementary material for: Competition for calnexin binding regulates secretion and turnover of misfolded GPI-anchored proteins
Source: J Cell Biol. 2023 Sep 13;222(10):e202108160. doi: 10.1083/jcb.202108160 (PMC10499038; doi:10.1083/jcb.202108160)
Supplement: SourceData F4 — is the source file for Fig. 4. [file JCB_202108160_SourceDataF4.pdf]

FIGURE 4 A

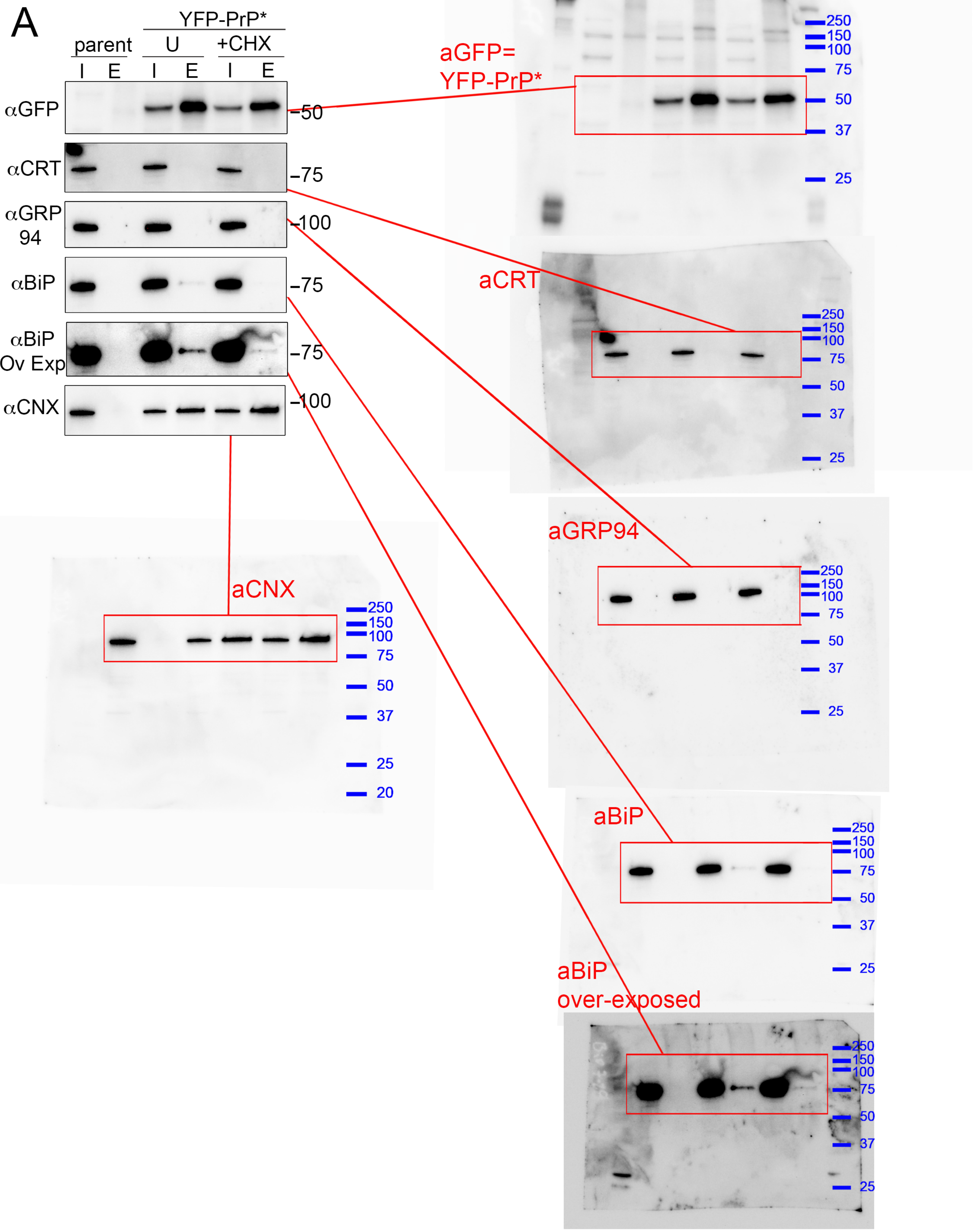
A
YFP-PrP*
parent
U
+CHX
I E I E I E
αGFP
αCRT
αGRP 94
αBiP
αBiP Ov Exp
αCNX
50
75
100
75
75
100
aGFP= YFP-PrP*
aCRT
aGRP94
aBiP
aBiP over-exposed
aCNX
250
150
100
75
50
37
25
20

FIGURE 4 E

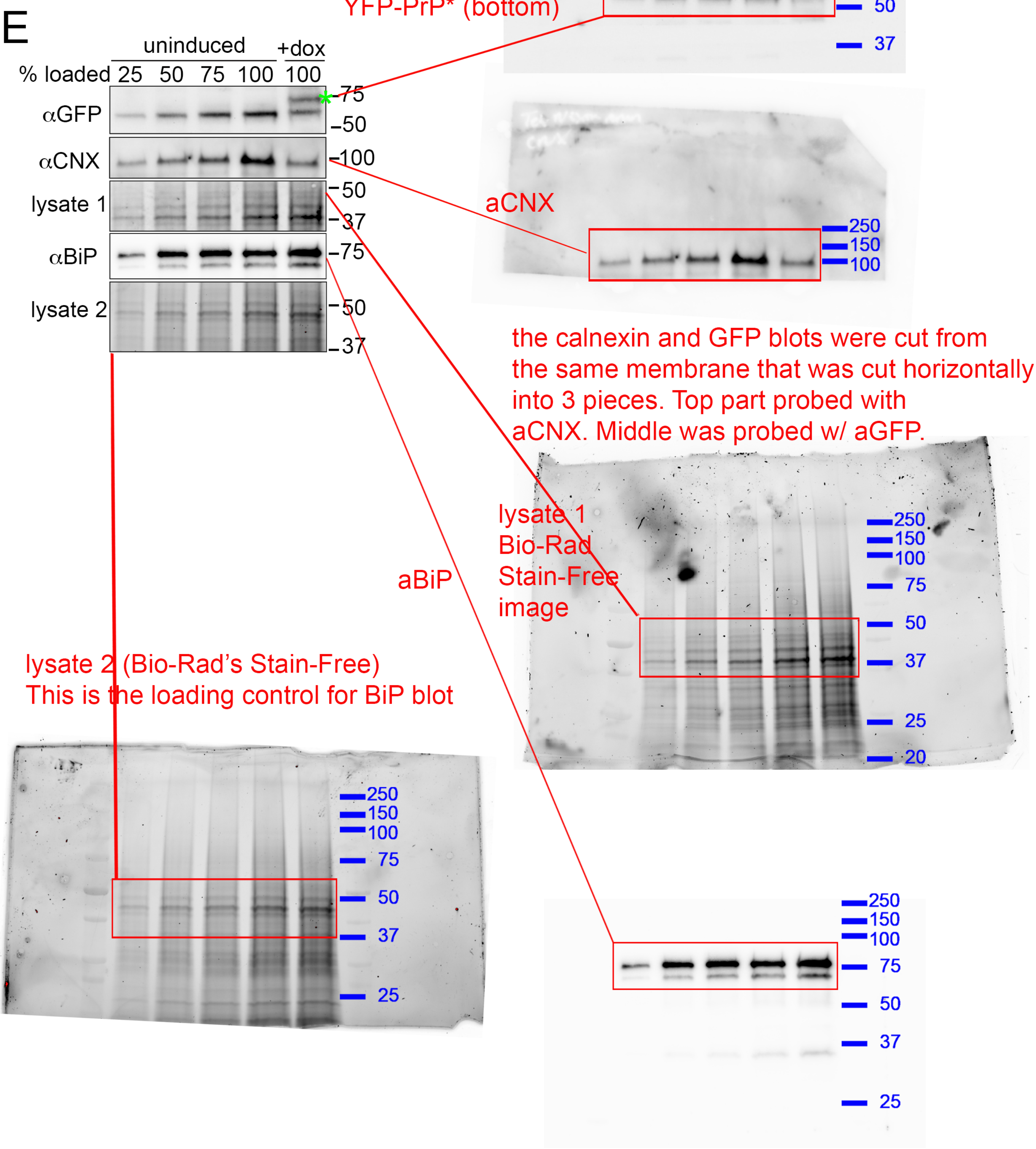
E
uninduced
+dox
% loaded 25 50 75 100 100
αGFP
αCNX
lysate 1
αBiP
lysate 2
75
50
100
50
37
75
50
37
aGFP=Cer-Nixin (top)
YFP-PrP* (bottom)
75
50
37
aCNX
250
150
100
the calnexin and GFP blots were cut from
the same membrane that was cut horizontally
into 3 pieces. Top part probed with
aCNX. Middle was probed w/ aGFP.
lysate 1
Bio-Rad
Stain-Free
image
250
150
100
75
50
37
25
20
aBiP
lysate 2 (Bio-Rad's Stain-Free)
This is the loading control for BiP blot
250
150
100
75
50
37
25
250
150
100
75
50
37
25

FIGURE 4 F

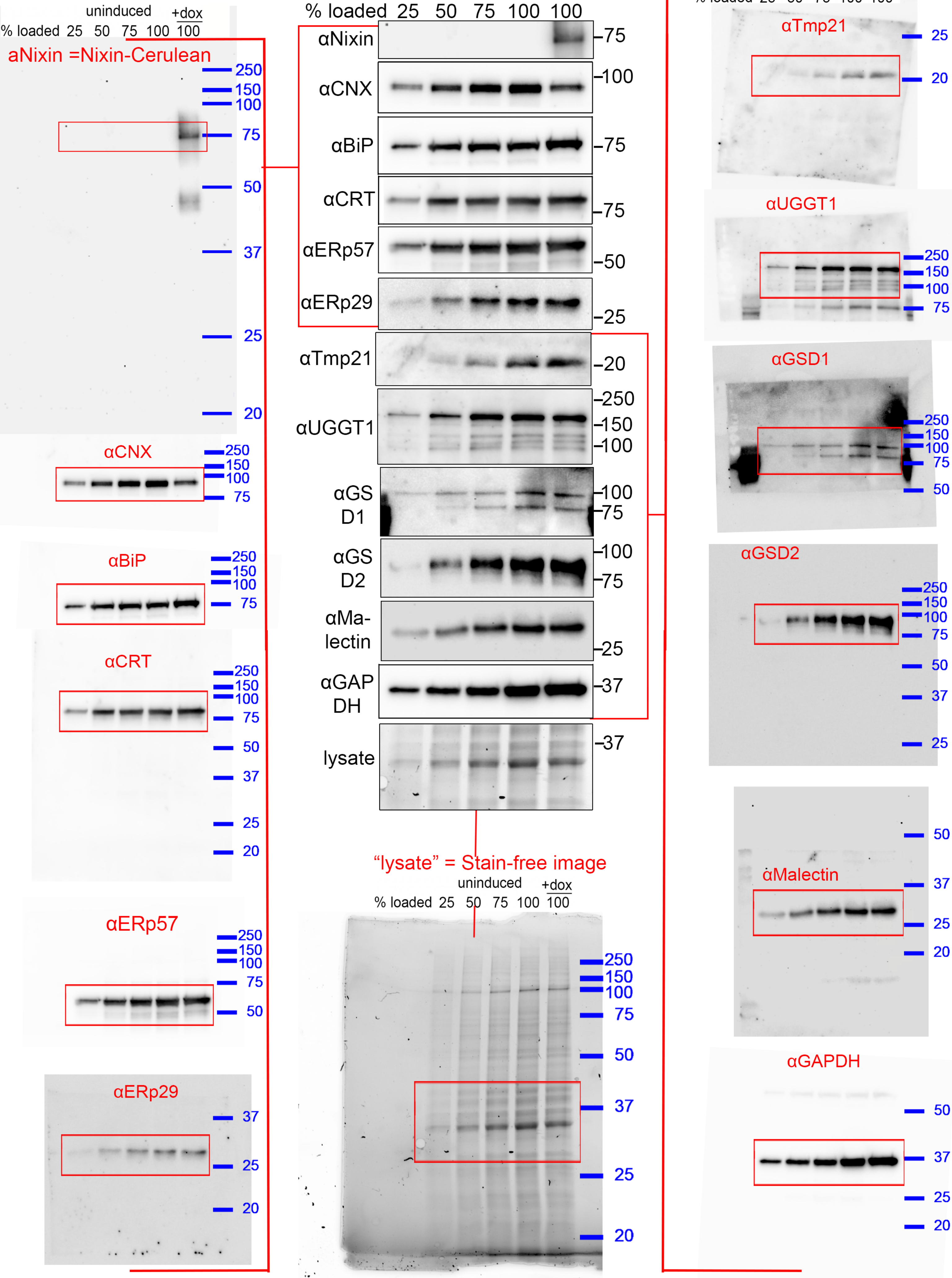

uninduced
+dox
% loaded 25 50 75 100 100
aNixin =Nixin-Cerulean
αNixin
αCNX
αBiP
αCRT
αERp57
αERp29
αTmp21
αUGGT1
αGS D1
αGS D2
αMa- lectin
αGAP DH
lysate
αGSD1
αGSD2
αMalectin
αGAPDH
"lysate" = Stain-free image
250
150
100
75
50
37
25
20
