# Supplementary figures and images for: Competition for calnexin binding regulates secretion and turnover of misfolded GPI-anchored proteins
Source: J Cell Biol. 2023 Sep 13;222(10):e202108160. doi: 10.1083/jcb.202108160 (PMC10499038; doi:10.1083/jcb.202108160)

FIGURE 5 C

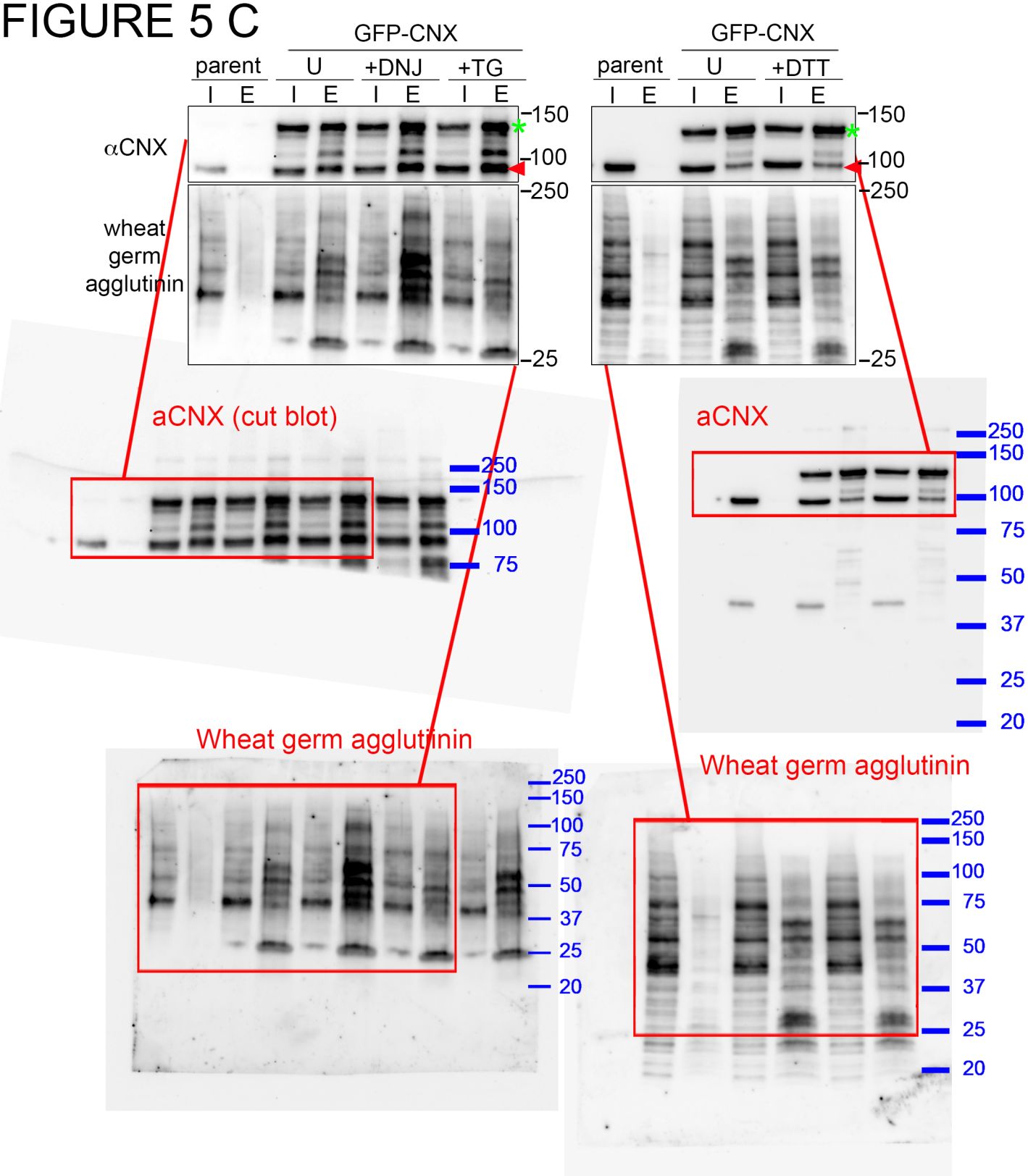

# FIGURE 5 D & E

## D

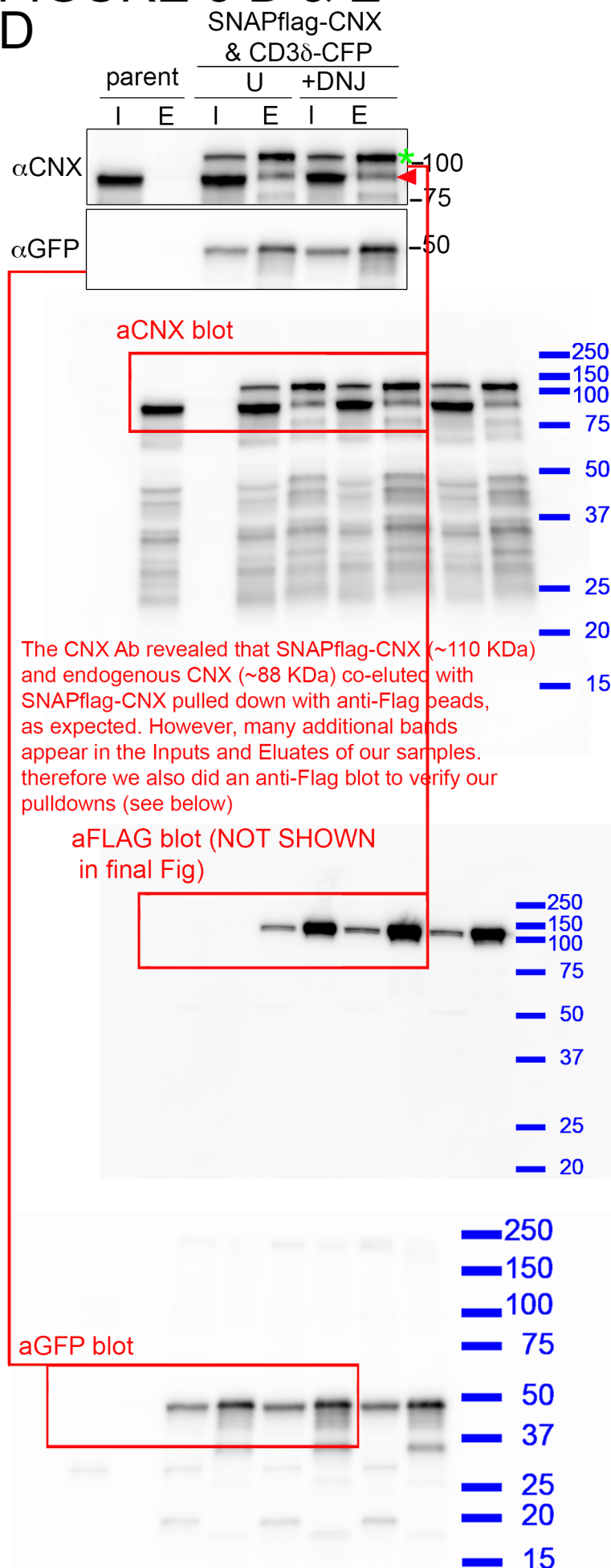

## E

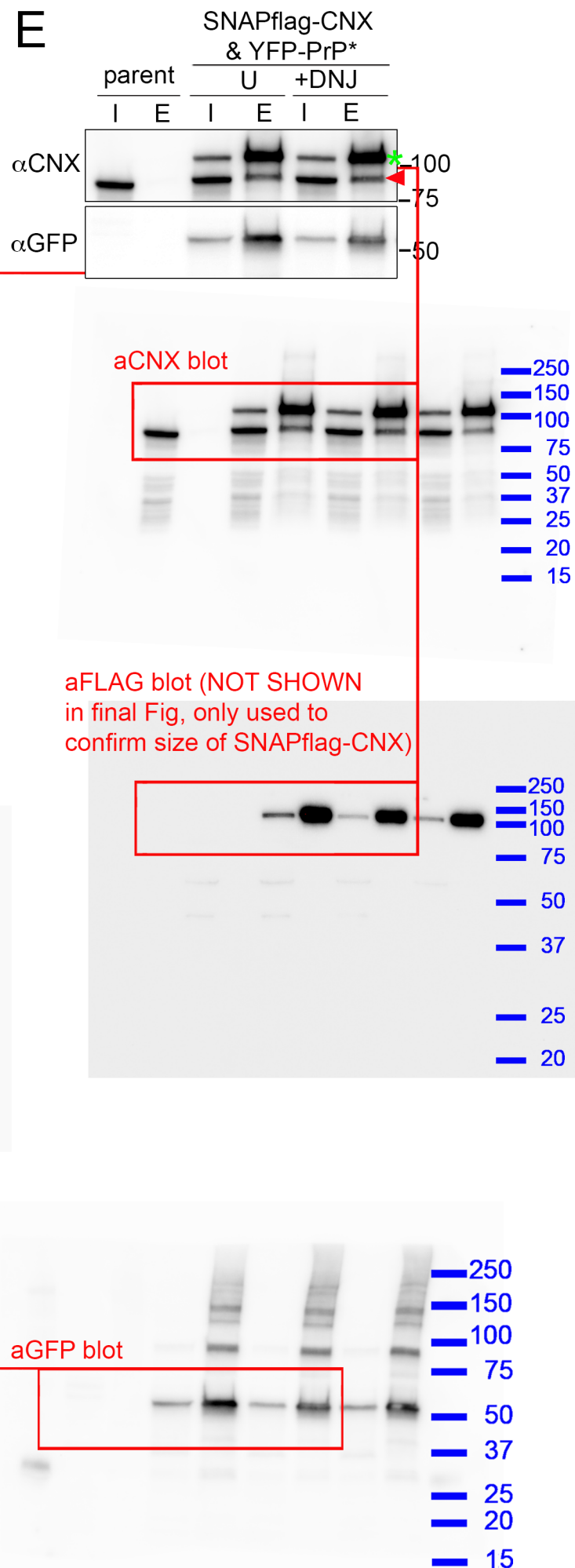

Supplement: SourceData F5 — is the source file for Fig. 5. [file JCB_202108160_SourceDataF5.pdf]

FIGURE 7 A, D, E and F

A

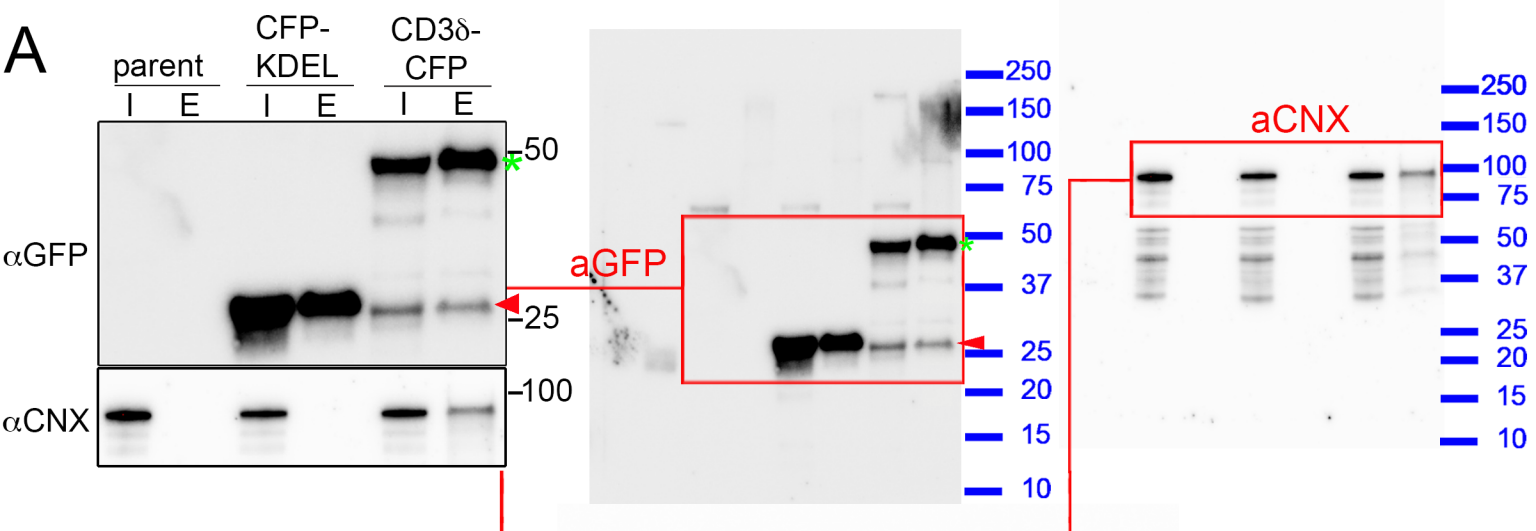

D

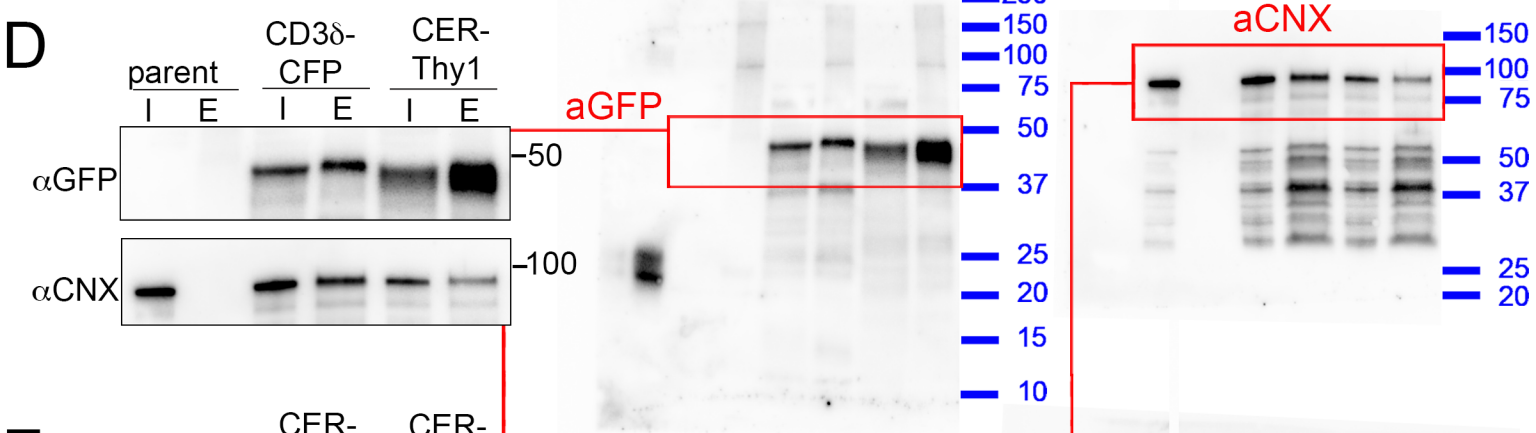

E

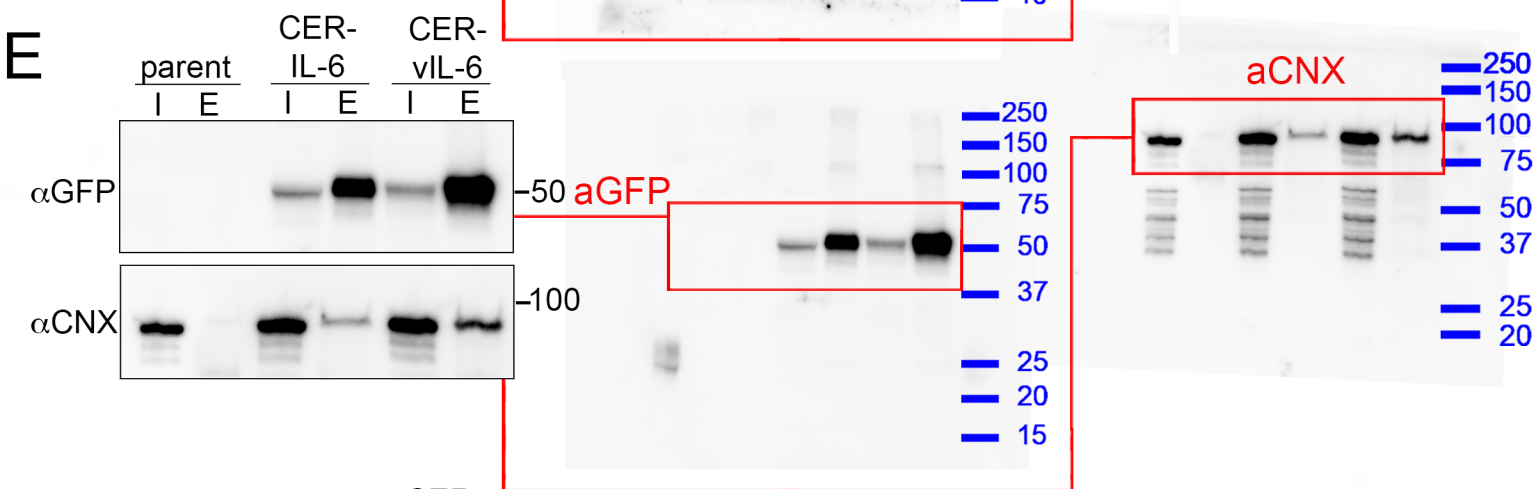

F

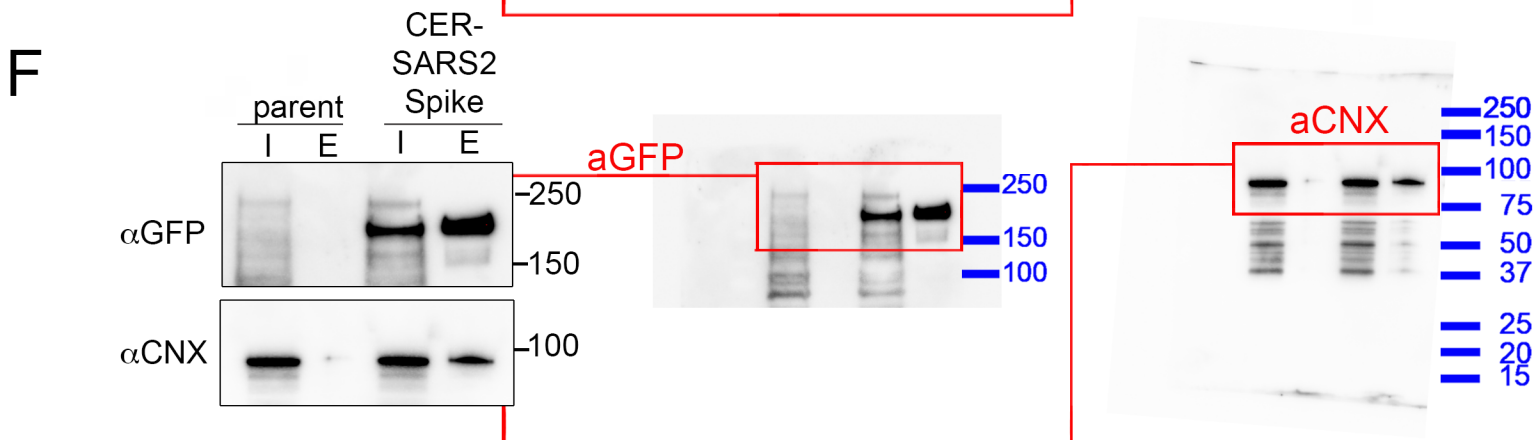

Supplement: SourceData F7 — is the source file for Fig. 7. [file JCB_202108160_SourceDataF7.pdf]

FIGURE 8 A

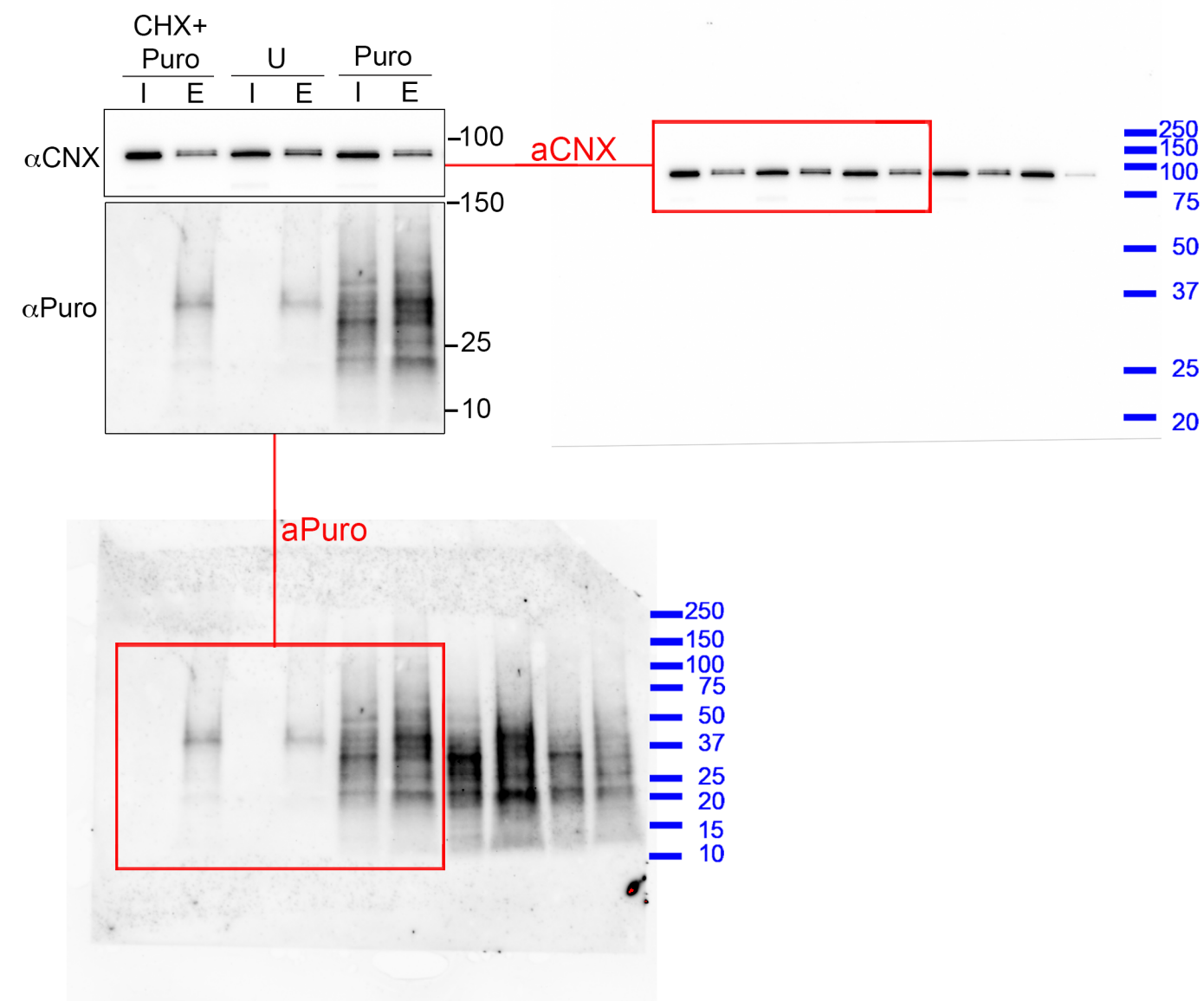

Supplement: SourceData F8 — is the source file for Fig. 8. [file JCB_202108160_SourceDataF8.pdf]

# FIGURE 9 C

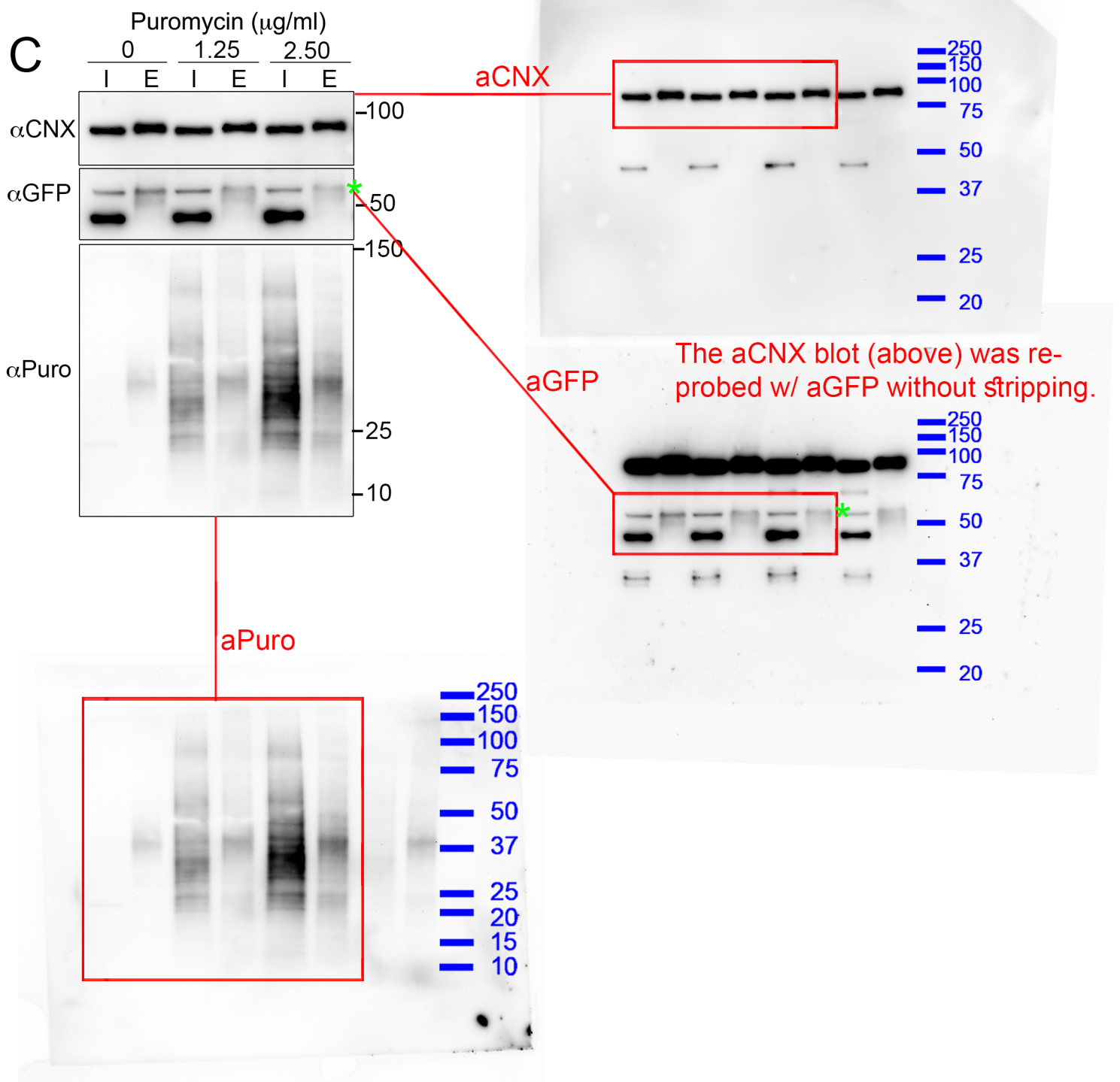

Supplement: SourceData F9 — is the source file for Fig. 9. [file JCB_202108160_SourceDataF9.pdf]

# SUPP FIGURE S 2 A

A

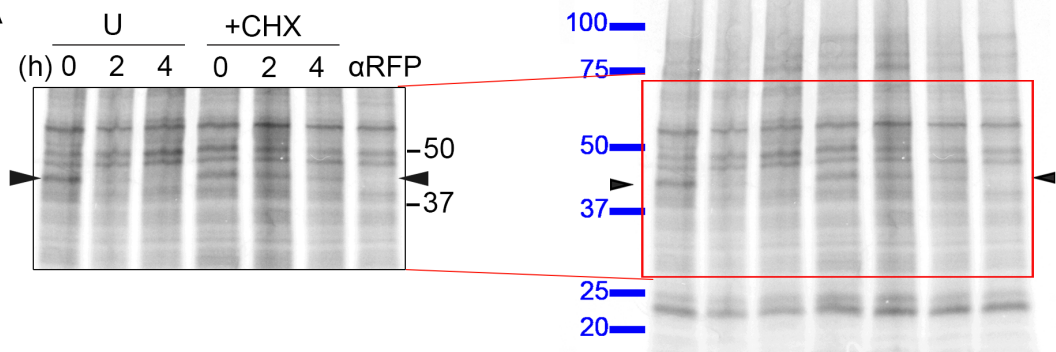

Supplement: SourceData FS2 — is the source file for Fig. S2. [file JCB_202108160_SourceDataFS2.pdf]
