# Supplementary material for: Competition for calnexin binding regulates secretion and turnover of misfolded GPI-anchored proteins
Source: J Cell Biol. 2023 Sep 13;222(10):e202108160. doi: 10.1083/jcb.202108160 (PMC10499038; doi:10.1083/jcb.202108160)
Supplement: SourceData FS1 — is the source file for Fig. S1. [file JCB_202108160_SourceDataFS1.pdf]

SUPP FIGURE S 1 A

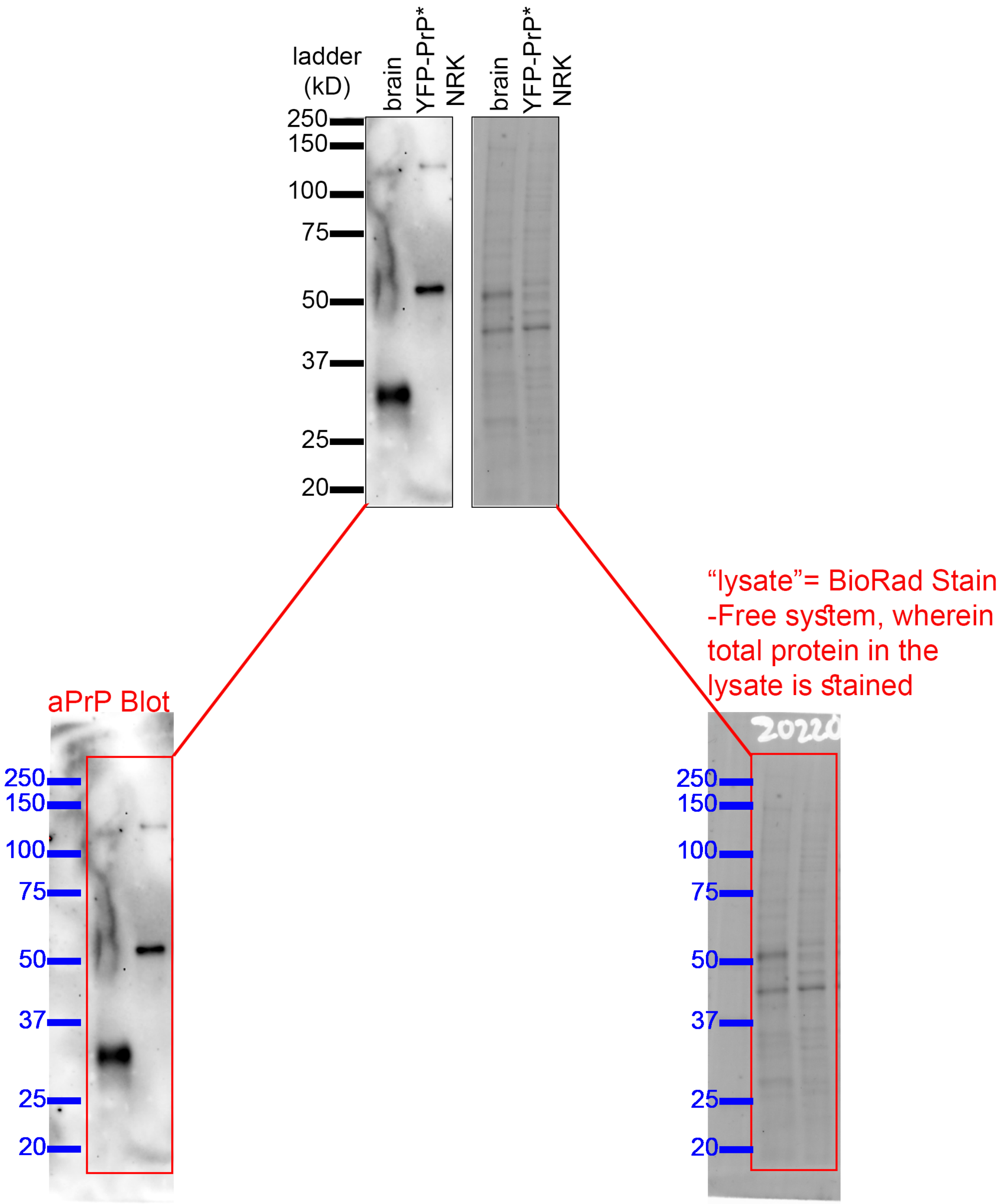

vertically cut strip from a larger membrane. I don’t have the remainder of this membrane, which was used for separate pilot experiments.
